# Large-scale transcriptomic analyses of major depressive disorder reveal convergent dysregulation of synaptic pathways in excitatory neurons

**DOI:** 10.1038/s41467-025-59115-4

**Published:** 2025-04-28

**Authors:** Fernando S. Goes, Leonardo Collado-Torres, Peter P. Zandi, Louise Huuki-Myers, Ran Tao, Andrew E. Jaffe, Geo Pertea, Joo Heon Shin, Daniel R. Weinberger, Joel E. Kleinman, Thomas M. Hyde

**Affiliations:** 1 Department of Psychiatry and Behavioral Sciences, Stanley and Elizabeth Star Precision Medicine Center of Excellence in Mood Disorders, Johns Hopkins School of Medicine, Baltimore, MD USA; 2 Department of Mental Health, Johns Hopkins Bloomberg School of Public Health, Baltimore, MD USA; 3 The Lieber Institute for Brain Development, Baltimore, MD USA; 4 Department of Biostatistics, Johns Hopkins Bloomberg School of Public Health, Baltimore, MD USA; 5 Department of Psychiatry, Johns Hopkins School of Medicine, Baltimore, MD USA; 6 Department of Neuroscience, Johns Hopkins School of Medicine, Baltimore, MD USA; 7 Department of Neurology, Johns Hopkins School of Medicine, Baltimore, MD USA; 8 McKusick-Nathans Institute of Genetic Medicine, Johns Hopkins University School of Medicine, Baltimore, MD USA

**Keywords:** Depression, Molecular neuroscience

## Abstract

Major Depressive Disorder (MDD) is a common, complex disorder that is a leading cause of disability worldwide and a significant risk factor for suicide. In this study, we have performed the largest molecular analysis of MDD in postmortem human brains (846 samples across 458 individuals) in the subgenual Anterior Cingulate Cortex (sACC) and the Amygdala, two regions central to mood regulation and the pathophysiology of MDD. We found extensive expression differences, particularly at the level of specific transcripts, with prominent enrichment for genes associated with the vesicular functioning, the postsynaptic density, GTPase signaling, and gene splicing. We find associated transcriptional features in 107 of 243 genome-wide significant loci for MDD and, through integrative analyses, highlight convergence of genetic risk, gene expression, and network-based analyses on dysregulated glutamatergic signaling and synaptic vesicular functioning. Together, these results provide an initial mechanistic understanding of MDD and highlight potential targets for novel drug discovery.

## Abstract (web summary)

Major Depressive Disorder (MDD) is a heritable psychiatric disorder whose biological basis remains poorly understood. This large-scale postmortem brain study identified extensive gene expression changes associated with genetic risk, highlighting disrupted glutamatergic signaling and synaptic vesicle function in MDD.

## Introduction

Major Depressive Disorder (MDD) affects 10–20% of individuals over their lifetime, and is a leading cause of morbidity, disability, premature mortality, and suicide^1^. While moderately effective psychotherapeutic and psychopharmacological treatments exist, a substantial proportion of individuals with MDD show limited response to available treatments, highlighting the pressing need to discover more rapid and effective treatments, particularly those targeted to the disorder’s underlying pathophysiology. Like other common psychiatric disorders, MDD has a major heritable component, with twin-based estimates of ~37% heritability in subjects with recurrent forms of MDD^2^. This significant heritability provides impetus to study genetic risk factors to identify mechanistic insights that may also provide therapeutic insights. However, while recent genome-wide association studies have discovered several hundred associated loci^3,4^, the heritability explained remains modest, and specific mechanisms by which the significant loci increase risk remain to be elucidated.

In contrast to large-scale genetic studies of MDD^3,4^, gene expression studies have been more limited in scope, whether conducted in the primary organ of interest or in surrogate tissues such as whole blood^5^. Postmortem brain studies of MDD, in particular, have been limited by small sample sizes (Supplementary Table 1), which are generally an order of magnitude smaller than analogous studies of other major psychiatric disorders^6^, and are likely to be underpowered given the expectation of small effect sizes in a highly polygenic and etiologically complex disorder such as MDD. An additional challenge of existing postmortem studies is the variability in brain regions studied^7–9^, and the variable consideration of potential confounders, both of which have likely contributed to the lack of converging results.

The largest study of gene expression in MDD reanalyzed microarray data from the prefrontal cortex in 87 subjects with MDD^10^. This study identified a gene co-expression module upregulated in MDD subjects that was enriched for G-protein signaling and cytokine-related interactions. However, more recent RNA-seq studies have been smaller in scale and varied in their analytical approaches, particularly in regard to controlling for experimental confounds, with limited evidence of replication (Supplementary Fig. 1). Emerging studies have also begun to utilize single-nucleus RNA sequencing (snRNA) technology to characterize cell-specific gene expression in postmortem brain. An initial study of the dorsolateral prefrontal cortex (DLPFC) in subjects with MDD analyzed 17 male cases and 17 controls, finding the largest number of differentially expressed genes in immature oligodendrocyte precursor cells and excitatory neurons^11^. A subsequent study of the DLPFC by the same investigators performed snRNA assays in a complementary sample 20 female cases with MDD and 18 female controls^12^. In both sex-specific and a combined meta-analysis of both studies, the highest number of differentially expressed genes was found in a deep-layer excitatory and microglial cell cluster, which were downregulated and upregulated in MDD cases, respectively.

Most studies of MDD have focused on the prefrontal cortex, leaving aside substantial evidence that emotional regulation has important correlates with activity in subcortical regions and in the cingulate cortex, which forms part of complex interconnected regions termed the “limbic system” by Paul McLean in the mid-twentieth century and is thought to play a major role in mammalian emotional behavior^13^. Moreover, mechanistic theories of emotional dysregulation and major depression have lacked consensus and validation^14^. The most prominent hypotheses have been based on “reverse translation” of the presumed therapeutic mechanism of antidepressants (monoaminergic dysfunction) and on molecular studies from animal models of chronic stress and stress response (neurotrophic hypothesis). However, these hypotheses have been challenging to evaluate directly in humans, and they have generally not been validated by human genetic studies, where the broad degree of polygenicity implicates substantial mechanistic heterogeneity and the need for more comprehensive approaches to etiological investigation.

In the current study, we have performed a well-powered study of gene expression in the brains of individuals with a history of recurrent MDD, utilizing high-depth RNA sequencing to uncover transcriptomic patterns associated with genetic risk for MDD and with the clinical state of the disorder. We have focused on two brain regions (the subgenual Anterior Cingulate Cortex [sACC] and the amygdala) highly associated with emotional processing in both animal models^15^ and human imaging studies^16,17^.

## Results

We performed RNA sequencing of 846 samples from 458 subjects (242 cases with recurrent MDD and 216 controls) across two brain regions implicated in emotional processing and MDD pathophysiology—the sACC and amygdala. The sample distribution was similar for sACC (228 cases and 200 controls) and the amygdala (231 cases and 187 controls). Our sample was predominantly male (*N* = 72.3%) and had an average age at death of 47.7 years (±0.6 SEM) [subject characteristics shown in Supplementary Dataset 1]. Cases had substantially higher polygenic risk scores of MDD, with an effect size consistent with more severe, clinically ascertained samples (pseudo-*R*^2^ = 6.3%, *P* = 3.0 × 10^−6^), and no evidence of differences in polygenic risk by sex (interaction *P* = 0.8) [Supplementary Fig. 2].

Using statistical methods for cellular deconvolution (see “Methods”)^18^, we observed differences in cellular proportions between the sACC and the amygdala, but few differences between MDD cases and controls (Supplementary Dataset 2, Supplementary Fig. 3). We found slightly lower estimated proportions of microglial cells in MDD in both the sACC and amygdala, as well as modestly higher proportions of inhibitory neurons and oligodendrocyte precursors in the sACC and amygdala, respectively. However, these differences were small, with changes in predicted proportions of 1% or less across these cell types (Supplementary Fig. 3).

### Differential gene expression analyses

**Fig. 1:**
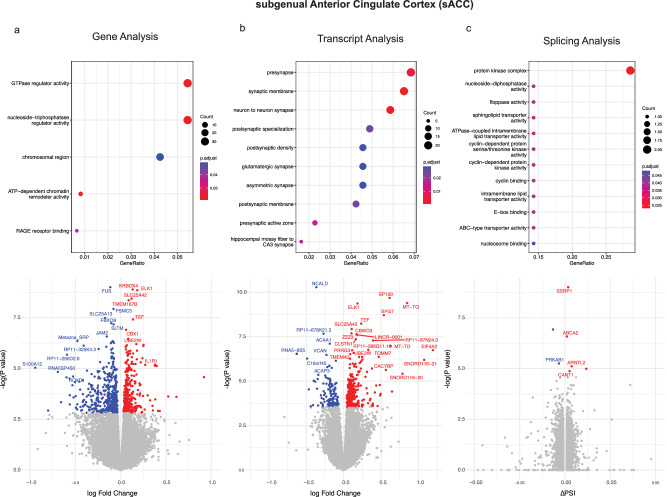
Differential expression and pathway enrichment results in the subgenual anterior cingulate cortex (sACC). Analyses are shown for differential (**a**) gene (**b**) transcript and (**c)** splicing results. Volcano plots display *log(fold change)* on the *x*-axis and the differential expression association *p* value on the *y*-axis. Genes with a false discovery rate (FDR)-corrected association *p* value < 0.05 are highlighted in red (upregulation) and blue (downregulation). Selected genes are labeled where possible without cluttering the plot. Above each volcano plot, the most significantly enriched GO ontology pathways are shown. Enrichment analyses were performed using the clusterProfiler R package with a one-sided (right-tailed) hypergeometric test, followed by FDR correction for the number of GO categories. Data for this figure are available in Supplementary Datasets 3 (differential expression) and 4 (GO enrichment).

All analyses were corrected for technical covariates, including RNA quality, alignment metrics, genotype-based principal components, and differential susceptibility to degradation, using experiment-based quality Surrogate Variable Analyses (qSVA) (see “Methods,” Supplementary Dataset 1 and Supplementary Figs. 4–7). Phenotype-based covariates such as antidepressant exposure, comorbid drug use, and mode of death were not significantly associated with variance in gene expression and, therefore, were not included as covariates (Supplementary Fig. 6). Primary analyses were performed at the level of the gene, splicing clusters, and transcripts using Gencode v25 annotations^19^. Combining all transcriptomic features, we found slightly more differentially expressed genes (FDR < 5%) in the sACC (*N* = 1192) compared with the amygdala (*N* = 936) [Fig. 1]. The average effect of significantly differentially expressed genes across the two brain regions was similar (1.11 fold change in the sACC and 1.13 in the amygdala) and comparable with similarly-sized studies of other adult psychiatric disorders^6,20^.

**Fig. 2:**
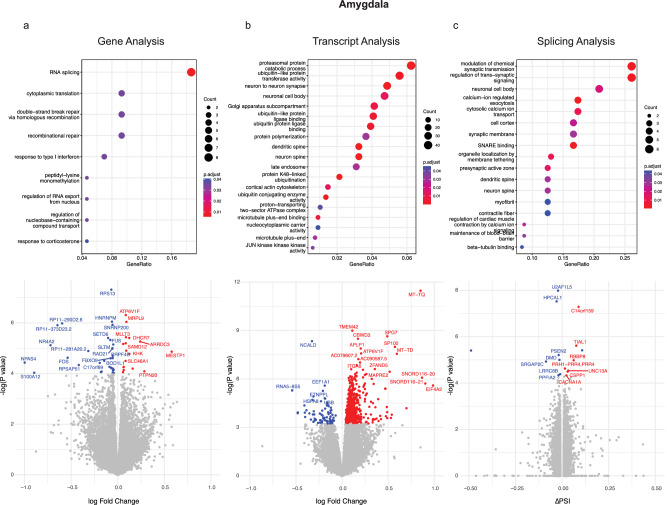
Differential expression and pathway enrichment results in the amygdala. Analyses are shown for differential (**a**) gene (**b**) transcript and (**c**) splicing results. Volcano plots display *log*_*2*_ *(fold change)*  on the *x*-axis and the differential expression association *p* value on the *y*-axis. Genes with an FDR-corrected association *p* value < 0.05 are highlighted in red (upregulation) and blue (downregulation). Selected genes are labeled where possible without cluttering the plot. Above each volcano plot, the most significantly enriched GO ontology pathways are shown. Enrichment analyses were performed using clusterProfiler with a one-sided (right-tailed) hypergeometric test, followed by FDR correction for the number of GO categories. Data for this figure are available in Supplementary Datasets 3 (differential expression) and 4 (GO enrichment).

At the level of the gene, we found 779 differentially expressed genes in the sACC and 50 in the amygdala at a genome-wide corrected FDR < 5% (Figs. 1 and 2, and Supplementary Dataset 3). Differentially expressed genes in the sACC included several calcium, potassium, and sodium channel voltage-gated subunits (*CACNA1B, CACNA1C, CACNA2D2, SCN4B, KCNIP4, KCNJ16, KCNJ2, KCNJ9, KCNQ2*), as well as subunits of excitatory (*GLUD1, GRM1*) and inhibitory (*GABRD, GAD2*) neurotransmitter genes. As shown in Supplementary Dataset 3, these excitatory and inhibitory genes were upregulated and downregulated, respectively. Gene ontology (GO) analyses of the significant DGE in the sACC (Fig. 1a, Supplementary Dataset 4) highlighted GTPase signaling, consistent with prior independent studies of cortical gene expression in MDD^10^. In contrast, the 50 differentially expressed genes in the amygdala were generally devoid of synaptic or neuronal genes, with the notable exception of *FOS* and *NPAS4*, two well-characterized neuronal activity-dependent immediate early genes involved in neuronal function and synaptic development that were downregulated in cases with MDD. Pathway analyses of the amygdala differentially expressed genes converged on gene splicing and protein metabolism/ubiquitination pathways along with genes in the response to inflammatory (interferon) and stress-related (corticoid) signaling (Fig. 2a). Across the sACC and amygdala, 20 DE genes were significantly associated in both regions, representing a significant enrichment (OR = 21.4, Fisher *P* < 2.2 × 10^−16^) compared with chance expectations (correlation shown in Supplementary Fig. 8a). These overlapping genes were notable for consisting of several downregulated genes associated with neurological disorders such as *FUS* (ALS)^21^*, ATXN10* (Spinocerebellar ataxia)^22^, and *NR4A2* (Parkinsonism)^23^.

### Differential transcript expression analyses

The use of alternative splicing to generate transcript diversity in the human brain^24^ may be particularly relevant a mediator of genetic risk for complex brain disorders^25^. We therefore performed transcript-level analyses using Salmon^26^, and found 438 differentially expressed transcripts in 406 genes in the sACC and 923 DTEs in 846 genes in the Amygdala (Supplementary Dataset 3), with an average effect size (fold change) of 1.12 and 1.10, respectively. Differentially expressed transcripts in the sACC showed strong enrichment and upregulation of synaptic genes (Fig. 1b, Supplementary Dataset 3) including widely implicated psychopathology genes such as the neuronal calcium channel subunit *CACNA1C*, and subunits of excitatory and inhibitory synapses such as the scaffolding protein SHANK2, the ionotropic glutamate receptor GRID1, the potassium channel subunits KCNQ2 and KCNMA1, and the presynaptic adhesion molecular NRXN3. Intriguingly, we also found upregulation of a transcript in Corticotropin-Releasing Hormone Receptor 1 (*CRHR1*), a central component of the HPA axis associated with animal models of depression and anxiety^27^.

Synaptic functioning also featured prominently among the differentially expressed transcripts of the amygdala (Fig. 2b, Supplementary Datasets 3 and 4), highlighting several synaptic proteins (NLGN1, SHANK2, SLITRK5, SNX9, SYT9, SYT11), calcium channel subunits (CACNG7, CACNA2D1), potassium channels (KCTD4/7/8/17, KCNA4, KCNB1, KCNJ9), as well as glutamate (GRIA2, GRIA4, GRM5) and GABAergic receptors (GABARAP, GABARAPL1). Similar to the gene-level analysis, we found strong evidence for overlapping transcripts expressed in the sACC and amygdala (*N* = 105 shared differentially expressed transcripts, OR = 30.3, Fisher’s exact test, *P* < 2.2 x 10^−16^ [see Supplementary Fig. 8b]).

### Differential splicing analyses

We used Leafcutter^28^ to identify differential splicing clusters within genes (see “Methods”). With an FDR threshold of 5% and an absolute intron percent splice in (ΔPSI) threshold of ≥1%, we identified 14 and 50 differentially spliced introns in 7 and 17 intron clusters in the sACC and amygdala, respectively (Figs. 1c and 2c and Supplementary Dataset 3). We found no overlap in associated clusters across the two brain regions, potentially also consistent with the greater regional specificity of splicing patterns compared to gene expression^24^, with mean differences in PSI values (ΔPSI) of 7.4% and 11.1 % in the sACC and amygdala, respectively.

Differentially spliced clusters (FDR < 5%) were few in the sACC (Fig. 1c) and notable for genes involved in splicing and transcriptional regulation (*SSRP1*)^29^, cholesterol transport, amyloid processing (*ABCA2*)^30,31^, and circadian rhythm functioning (*ARNTL2*)^32^. Similar to the transcript-based analyses, we found more differentially spliced clusters in genes within the amygdala, with pathway analyses converging on synaptic functioning, excitatory neurons, and calcium signaling (Fig. 2c, Supplementary Dataset 4). Among the synaptic genes are *PPFIA2*, a scaffolding protein located in the presynaptic active zone^33^, *SRGAP2C*, a human-specific GTPase associated with synaptic development in excitatory and inhibitory neurons^34^, and *Unc13*, an essential protein for synaptic vesicle release in excitatory neurons^35^ and a known risk gene for ALS and Frontotemporal Dementia^36^. Neuronal calcium signaling genes include *HPCAL1* (hippocalcin-like 1), a neuron-specific calcium-binding protein specific to layer 2/3 excitatory neurons^37^, and the calcium channel subunit CACNA1A, which has been associated with a broad array of neurological and cognitive phenotypes^38^. We also found differential splicing of *Presenilin 2* in the Amygdala, one of the most common genes associated with early-onset familial Alzheimer’s disease, with a well-known role in the production of amyloid beta. Interestingly, this association is with a splicing variant in exon 6 in *Presenilin 2*, which has been similarly reported in the brains of Alzheimer’s disease^39^. Finally, we also found an association in *DGLUCY* d-Glutamate cyclase, a protein^40^ in the mitochondria that degrades d-glutamate, an enantiomer of glutamate found at low levels in brain tissues^41^.

### Exploring the effect of expression differences by sex and mode of death

MDD is significantly more prevalent in women, and prior, but small, studies of gene expression have suggested significant sex differences in expression signatures between individuals with MDD and controls^7,42^. In our primary analyses, we controlled for sex as a covariate and focused on the study of transcriptional features associated with MDD common across men and women. To explore the hypothesis that gene expression changes may be sexually dimorphic, we repeated our differential expression analyses at the gene and transcript levels and tested for an interaction between sex and case-control status. As shown in Supplementary Dataset 5, we found very few genes or transcripts with significant FDR-corrected interaction terms (1 and 28 transcripts in the sACC, and 5 genes and 15 transcripts in the Amygdala). Moreover, none of these features with significant sex-based interactions had significant main effects in the differential gene or transcript expression analyses described above (Supplementary Dataset 3), nor did they have significant main effects in either males or females in exploratory post hoc sex-stratified analyses. While these findings do not exclude the possibility of sex-specific effects, they suggest that specific sex-associated effects are likely to be modest and will require much larger sample sizes to identify^43^.

MDD is a significant risk for suicidal behavior, with death by suicide being present in almost a third of our case samples (*N* = 69). As described earlier, mode of death did not contribute substantially to variance in gene expression (Supplementary Fig. 6). Nevertheless, to further explore transcriptional differences in subjects who died by suicide versus those who died by natural causes, we performed case-only analyses with suicide as the outcome variable. As shown in Supplementary Dataset 6, there were no gene or transcript-based associations in either brain region that remained significant after correcting for multiple testing. These results, similar to our sex-interaction analyses, suggest that the effect of suicide on our gene expression results is likely to be small.

### Integrative analyses of GWAS and QTL data

We conducted a series of GWAS/quantitative trait loci (QTL) based integrative analyses to identify genetically mediated gene expression using analytic frameworks that minimize the potential confounding from the effects of the illness or its treatment. We utilized three different integrative methods—Transcription-Wide Association Study (TWAS)^44^, co-localization^45^, and Summary Mendelian Randomization (SMR)^46^—that provide complementary approaches to infer potential targets of transcriptionally mediated genetic risk^47,48^.

**Fig. 3:**
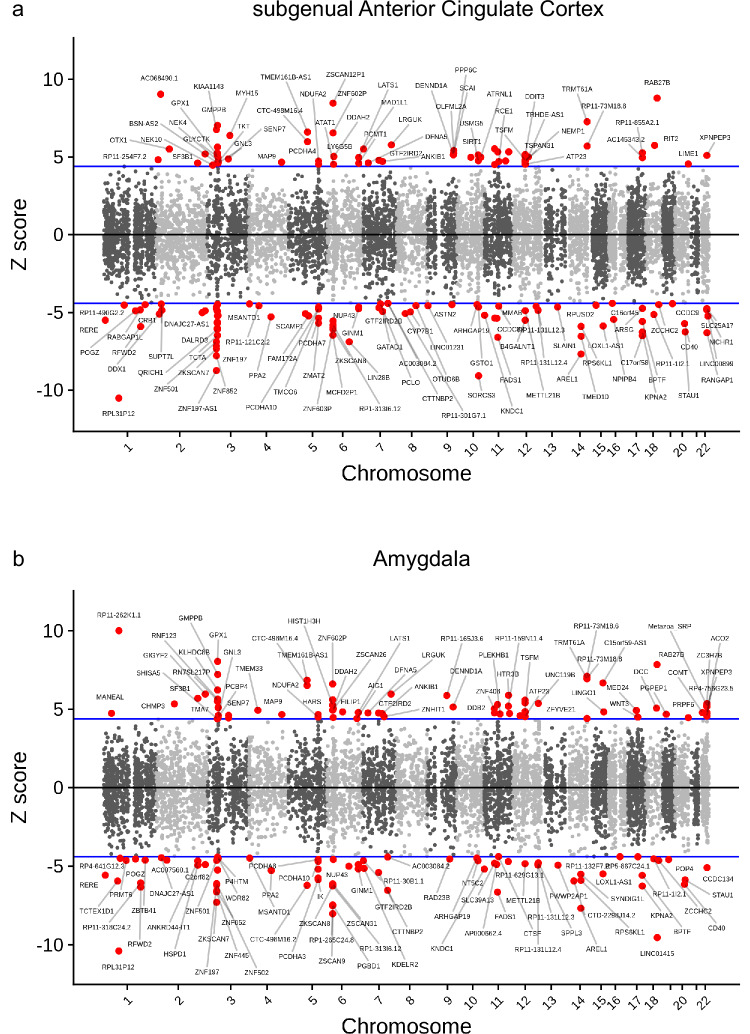
Transcription-Wide Association Study (TWAS) plots in the subgenual anterior cingulate cortex and amygdala. **(a)** sACC and (**b)** Amygdala. Each plot shows the predicted *z*-score (standardized effect size) per gene. The blue line indicates the threshold for Bonferroni correction based on the number of genes with significant expression-based heritability. Data for this figure are available in Supplementary Dataset 7.

First, we combined the results of the most recent MDD GWAS meta-analyses^3^ and our case-control transcriptome dataset to identify predicted levels of genetically mediated gene expression in the sACC and amygdala using the TWAS framework^44^. After adjusting for case-control status and selecting genes with a significant *cis-*based gene expression heritability, TWAS identified a total of 9170 genes with significant cis-based heritability (mean *h*^2^ = 0.17) in the sACC and 8477 (mean *h*^2^ = 0.16) in the amygdala. TWAS analyses subsequently revealed a similar number of genes significantly associated with MDD in the sACC (*N* = 135) and amygdala (*N* = 129) at Bonferroni-corrected significance levels (Fig. 3, Supplementary Dataset 7). The majority of these TWAS significant associations (75.4 %) were in GWAS-significant loci (defined as ±500 kb of the most associated index marker), with approximately half (*N* = 66) of TWAS-associated genes being significant in both the sACC and amygdala (enrichment *p* value < 2.2e-16). In the sACC, five genes were significant in both TWAS and differential expression analyses; these included *RIT2* and *RAB27B—*both GTPase-associated proteins involved in dendritic spine development and vesicular function, respectively, that have also been linked to autophagy of α-synuclein in cellular models of Parkinson’s disease^49,50^.

Second, we focused on the recently identified 243 MDD risk loci^4^ and performed QTL mapping, followed by co-localization^45^, and Mendelian randomization analyses^46^ of the genome-wide associated loci^4^. To increase power for eQTL identification, we combined our samples with a similarly performed study of bipolar disorder^51^, yielding a harmonized dataset of 551 independent samples in the sACC and 540 in the amygdala that represents the largest transcriptomic resource available for either brain region. After controlling for case-control status, experimental, and ancestry-based confounds (see “Methods”), we performed a cis-based QTL analysis at the level of the gene, local splicing clusters, and transcript^52^ (data provided in https://zenodo.org/records/13906097). As expected, QTLs were strongly enrichmed at the transcription start site (Supplementary Fig. 9).

**Fig. 4:**
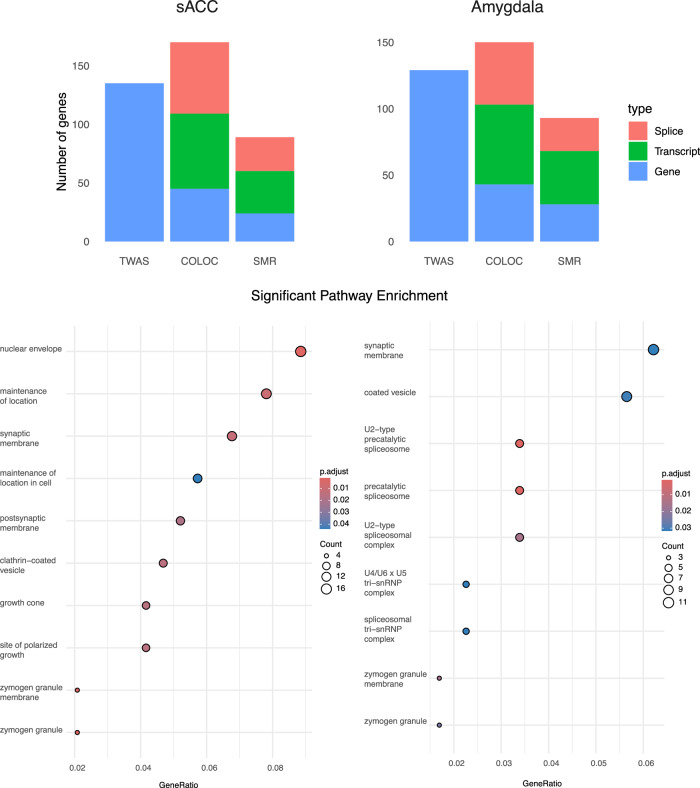
Distribution of TWAS/QTL-associated features in MDD loci in the sACC and amygdala. The top panel shows the distribution of TWAS/QTL-associated features in MDD loci. The bottom panel shows significantly enriched Gene Ontology (GO) pathways. Enrichment analyses were performed using clusterProfiler with a one-sided hypergeometric test, followed by FDR correction for the number of GO categories.

At the level of the gene (Supplementary Datasets 8 and 9), we found 59 genes with significant GWAS/QTL associations (based on coloc and/or SMR analyses) in the sACC and amygdala, with 36 genes associated in both brain regions. These included the well-established pleiotropic genes *FURIN*, *FADS1*, as well as several postsynaptic density (PSD) proteins, including *CTTNBP2*, *GABBR1*, *NEGR1*, *SEMA3F*, and *KPNA2*. Compared to these gene-based analyses, we found significantly more associations with local splicing-based transcriptional features (Fig. 4, Supplementary Datasets 8 and 9), highlighting the importance of splicing variation as putative mediators of genetic risk for MDD^53^. There were 89 genes linked to splicing QTLs in MDD loci, 48 of which were only found through splicing QTLs. Notable splicing QTLs in both sACC and the amygdala included  the following synaptic genes: *NRXN1, GABRA1*, the RNA-binding proteins *RBFOX*, and the calcium channel subunit *CACNA1C*. Finally, in our transcript-based integrative analyses, we identified 111 genes with associated transcripts, of which 43 were not detected in our gene-based or splicing QTL-based analyses. Significant transcript-QTLs (Fig. 4, Supplementary Datasets 8 and 9) found to be associated with GWAS loci in both the sACC and amygdala include those in Neuroligin 1 (NLGN1), Vesicle-associated membrane protein 2 (*VAMP2*), Huntingtin (*HTT*), and *SLC12A5*, a neuron-specific potassium-chloride cotransporter broadly involved in GABA signaling^54,55^ and cortical development^56^.

Overall, these analyses revealed both independent and convergent associations across different transcriptional features (see Supplementary Fig. 10a, b and Supplementary Datasets 7–9). QTL/GWAS associations at the transcript level showed the most overlap both with local spicing and gene-based features (Supplementary Fig. 10), including several notable genes (*CHMP3*, *RAB27B*, *RFWD2*) with significant associations in all three transcriptional features (gene, local splicing, and transcript). When considering all integrative methods (TWAS, coloc, and SMR), we found associated transcriptional features in 107 of 243 MDD loci (101 in the sACC and 84 in the amygdala), with the majority (*N* = 79) being associated in both brain regions. Pathway enrichment analyses of these GWAS loci-associated transcriptional features were notable for both synaptic and vesicular ontology terms in both sACC and amygdala, and a more prominent role of splicing regulation in the amygdala (Fig. 4).

### Network analyses link genetic risk and transcriptional dysregulation to synaptic activity and alternative splicing in excitatory neurons

**Fig. 5:**
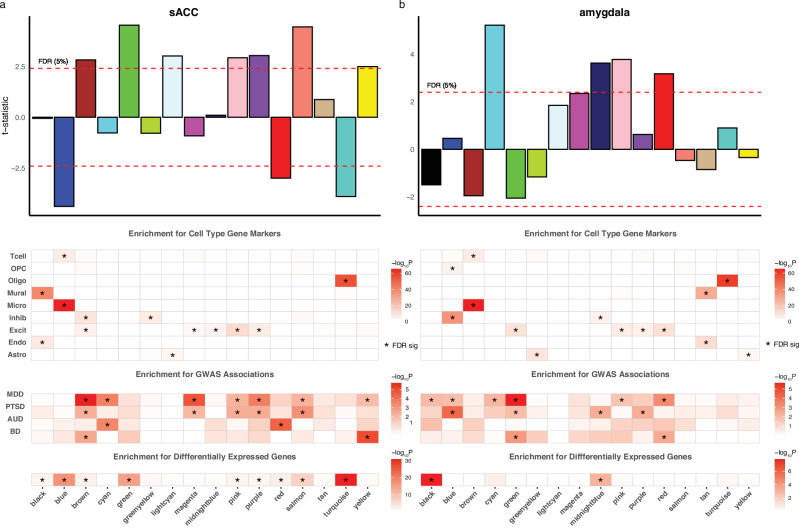
Network analyses and enrichment across CNS cell types, genetic risk, and differentially expressed genes. Weighted Gene Co-expression Network Analyses (WGCNA) in (**a**) the subgenual Anterior Cingulate Cortex and (**b)** the Amygdala. Modules are labeled alphabetically, with association *t*-statistics on the *Y*-axis representing the strength of each module eigengene’s association with case-control status. The dotted line represents the FDR < 5% threshold. The heatmaps (lower panels) show specific enrichment for cell-type-specific expression profiles, MDD, PTSD, Alcohol Use Disorder (AUD), and Bipolar Disorder (BD) GWAS-based gene (MAGMA) analyses, along with differentially expressed genes from the current study. Enrichment *p* values (two-sided) that meet FDR < 5% are marked with stars in the relevant heatmaps.

To identify further insights from transcriptional network analysis, we performed weighted gene co-expression network analyses (WGCNA) on our covariate-corrected gene expression dataset. Co-expression networks were constructed separately for the sACC and amygdala, with each identifying 16 modules ranging from 90 to 1524 genes (see Supplementary Dataset 10, Fig. 5, and “Methods”). All identified modules showed robust enrichment for protein-protein interaction networks (Supplementary Dataset 10), with the majority of modules showing specific associations with the major CNS cell types (Fig. 5a, b).

In the sACC, we found significant associations with the expression-based eigengenes of 10 modules and MDD case-control status, seven of which were also enriched for genes identified by the GWAS meta-analysis of MDD (Fig. 5a)^4^. The most strongly upregulated module (green) was notably enriched for pathways involving synaptic regulation and GTPase activity (Supplementary Dataset 10), aligning closely with the enrichment in our DE results (Fig. 1a) and replicating previous findings from a microarray-based networks study of MDD^10^. Among the seven modules upregulated in the MDD case-control analysis, six were strongly enriched for synaptic and neuronal pathways. Three of these modules (brown, pink, and purple) additionally exhibited enrichment for glutamatergic and mixed glutamatergic/GABAergic single-cell expression signatures and contained GWAS-associated genes. This synaptic, glutamatergic, and mixed excitatory/inhibitory expression profile suggests a potential causal role for these pathways and cell types in MDD etiology. In contrast, three significantly downregulated modules were enriched for differentially expressed genes, but did not show any enrichment for GWAS-associated loci, suggesting that these processes may be instead associated with the lifetime consequences of MDD. Unlike the neuronal and synaptic pathways found in the upregulated modules, these modules were characterized by inflammatory (blue), myelination (turquoise), and splicing (red) pathways.

Network analyses of the amygdala revealed similar enrichment for the major CNS cell types and identified significant positive associations between MDD and the eigengenes of four modules (Supplementary Dataset 10, Fig. 5b). All four modules demonstrated enrichment for synaptic pathways, with one of the four modules (red) also showing enrichment in GTPase activity (Supplementary Dataset 10). Notably, this red module also exhibited significant enrichment for GWAS-associated loci, including several RNA-binding genes, *RBFOX1*, *CELF2*, and *CELF4*, as well as well-characterized presynaptic (*BSN*, *PCLO*) and glutamatergic (*GRM5*, *GRIK5*) genes. Three of these four MDD-associated modules also showed enrichment for markers of excitatory and inhibitory neuronal cells, highlighting a similar pattern of genetic risk and transcriptional dysregulation in glutamatergic and GABAergic cells that was observed in the sACC analyses.

Finally, to contextualize these findings within the broader spectrum of psychiatric disorders commonly comorbid with depression, we performed enrichment analyses for genes identified by GWAS meta-analyses of post-traumatic stress disorder (PTSD), alcohol use disorder, and bipolar disorder in both sACC and amygdala. As illustrated in Fig. 5a, b, we observed a similar pattern of genetic enrichment for PTSD across WGCNA modules, whereas enrichment patterns for alcohol use disorder, and particularly bipolar disorder, were more divergent.

### Convergence of GWAS-associated QTLs and differentially expressed features in synaptic genes

**Fig. 6:**
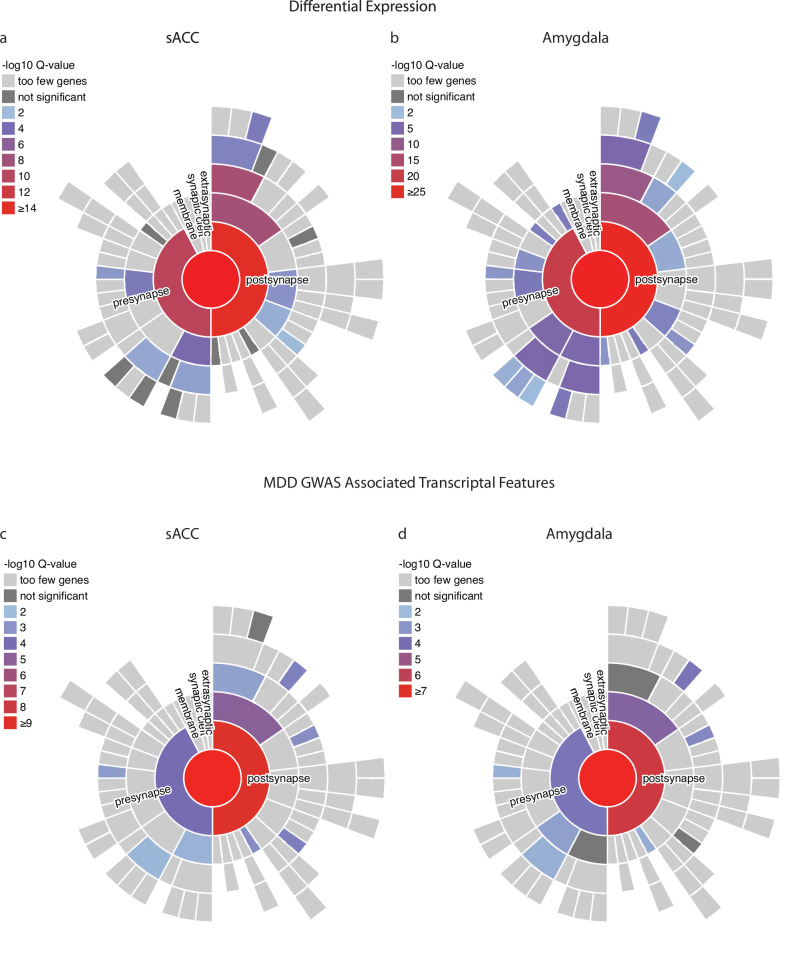
Synaptic enrichment of differentially expressed and genetically mediated associations. **a**, **b** Synaptic Gene Ontology enrichment plots for combined differential expression results in the sACC and Amygdala. **c**, **d** Combined QTL features in the sACC and Amygdala, respectively. Enrichment for each SynGO term was tested using a one-sided Fisher’s exact test followed by FDR correction for multiple testing.

Given the strong enrichment of neuronal cell-expressed genes and synaptic pathways in our prior analyses, we used the SynGO synaptic ontology database^57^ to further explore the role of the synapse in both our differentially expressed (gene, transcript, and splicing features) and QTL-based (gene, transcript-based, and splice QTLs) analyses. As shown in Fig. 6a, b and Supplementary Dataset 11, our gene expression data exhibited strong enrichment for both presynaptic and postsynaptic categories, with strikingly similar patterns evident in both the sACC and amygdala. Although overlap at the individual transcriptional feature level was uncommon, we similarly identified enrichment of presynaptic and postsynaptic genes harboring QTL-based associations in both brain regions (Fig. 6c, d). These results provide complementary evidence of a broad “synaptogenic hypothesis of depression,” initially proposed from stress-based animal models and now corroborated by human postmortem studies^58^.

## Discussion

In this study, we present the findings from the largest postmortem human brain transcriptomic study of MDD performed to date, focusing on two brain regions closely linked to the pathophysiology^16,59,60^ and treatment of MDD^61,62^. Our results revealed a significant enrichment for pre- and postsynaptic pathways, along with enrichment in previously implicated pathways associated with gene splicing and GTPase signaling. Notably, synaptic proteins are the primary targets of virtually all known antidepressants, including novel rapidly-acting agents^63^. While splicing and GTPase signaling serve broader canonical cellular functions, they also have a central regulatory role in regulating dendritic spines and synaptic plasticity^64,65^.

In further analyses mapping of MDD-associated genome-wide loci, we find significant associations in 107 of 243 GWAS loci across both brain regions, with similar enrichment in vesicular and synaptic pathways.

A key finding of our study, along with emerging human postmortem studies^6,66^, is the substantial number of differentially expressed and genetically mediated associations in splicing and transcript-based analyses. These results highlight the critical role of alternative splicing and isoform diversity in bridging genetic risk and transcriptomic variation in psychiatric disorders such as MDD. However, consistent with prior studies^20,51,67^, we observed limited overlap between differentially expressed features and those identified in QTL-based integrative analyses. This limited overlap likely results from insufficient statistical power to detect subtle QTL effects due to minor differences in risk allele frequency between cases and controls, as well as the complex cumulative impact of genetic risk, environmental influences, homeostatic responses, and lifelong treatment^68^ that can emerge in case-control analyses. Nevertheless, we still find significant convergence at the level of the pathway, with both our differential and QTL-based analyses on vesicular and synaptic pathways.

Among significant genes, *RAB27B* exhibited the strongest and most consistent associations across analytical approaches and brain regions, showing upregulation in both differential expression and TWAS analyses, and highlighted by integrative analyses (Coloc and SMR) through multiple splicing- and transcript-based QTLs in both sACC and amygdala.

*RAB27B* is a neuronally expressed GTPase involved in excitatory synaptic vesicle trafficking^69^ and in the localization of TrkB, the receptor for brain-derived neurotrophic factor, to the axonal membrane^70^. Additional genes involved in synaptic neuroplasticity include the proteins cortactin binding protein 2 (*CTTNBP2*), *SHANK2* (also known as Cortactin Binding Protein 1), Neuroligin 1 (*NLGN1*), and the calcium channel subunit *CACNA1C*. *CTTNBP2*, *SHANK2*, and *NLGN1* are cytoskeletal proteins found in the postsynaptic density, which have been previously associated with neurodevelopmental disorders and are key components of glutamatergic spine formation and synaptic neuroplasticity^71^. *CACNA1C*, one of the most consistently associated GWAS loci across mood and psychotic disorders, encodes an L-type voltage-gated calcium channel critical for neuronal excitability and synaptic function^72^.

The network (WGCNA) analyses further highlighted the important role of excitatory neurons in the pathophysiology of MDD, with significant evidence of upregulation of “eigengenes” in modules enriched for glutamatergic neurons and GWAS-associated genes (Fig. 5a, b). Identified in the analyses of both sACC and amygdala, these excitatory modules with converging genetic risk and gene expression were characterized by enrichment of several synaptic pathways, including regulation of synaptic vesicle exocytosis, glutamatergic signaling, and regulation of the PSD (Supplementary Dataset 10). In contrast, fewer modules were associated with inhibitory neurons, underscoring a potential excitatory-inhibitory imbalance frequently observed in animal models of chronic stress^73^, suggesting a significant causal role in MDD pathogenesis. Notably, modulation of glutamatergic signaling has emerged as a promising therapeutic target for novel antidepressants^74^.

Interestingly, our network analyses also found reduced expression of eigengenes associated with two glial modules in the sACC, representing microglial (blue module) and oligodendrocyte (turquoise module) cellular populations. Reduced levels of microglia-associated genes were similarly found in our earlier study of bipolar disorder^51^ and caution against overly simplistic hypotheses of increased inflammation in MDD. Instead, this downregulation could reflect disruption of microglia’s non-immune, pleiotropic roles, such as synaptic regulation^75^.

Consistent with comparable studies of other psychiatric disorders^10,20,51^, our results point to a broad number of modestly dysregulated genes consistent with what is now widely accepted to be a polygenic risk architecture in MDD. Although this study represents the largest transcriptomic analysis of MDD to date, the subtle effects observed in our differential expression analyses—coupled with the polygenic nature of MDD—suggest that even larger sample sizes will be required to comprehensively identify genes and networks associated with the disorder. Similarly, the substantial number of MDD-associated GWAS loci lacking clear colocalized transcriptomic features indicates that additional QTL discovery may further enhance the mapping of genetic risk in MDD. Indeed, limited statistical power remains an important constraint in our analysis, likely explaining the minimal overlap we observed between differential expression and QTL-based analyses, particularly given the modest differences in risk allele frequencies typical of common MDD-associated variants.

Importantly, while we used analytical methods to characterize and control for subtle differences in cellular heterogeneity, the broad tissue-level resolution of our gene expression data remains a significant limitation, as current deconvolution methods are imprecise and challenging to benchmark^76,77^. Single-nucleus RNA sequencing methods, which provide higher cellular resolution, are rapidly becoming standard; however, these methods remain costly for large samples, lack deep sequencing coverage per cell, and do not yet reliably offer transcript-specific resolution. Technological advances such as long-read sequencing are likely to soon overcome these limitations inherent in short-read RNA sequencing studies—including ours—which inevitably face challenges related to transcript assembly and accurate quantification^78^.

Additionally, we recognize the limitations of focusing on only two brain regions and  the absence of an age associated longitudinal design, which would be necessary for understanding the developmental profile of transcription dysregulation in MDD^14^. Our sample also included a higher proportion of males than typically observed in population-based surveys of recurrent MDD, reflecting the sampling practices inherent in procurement from the Medical Examiner’s office^79^. Although this male predominance reduces our power to detect sex-specific associations, sensitivity analyses confirmed that our primary findings were not substantially biased by this imbalance. Notably, despite these constraints, our study remains the largest transcriptomic analysis of MDD to date for both male and female subjects.

Finally, given the frequent comorbidities associated with psychiatric conditions like MDD, our results may still be influenced by residual confounds such as co-occurring disorders, lifetime substance use, medication effects, and the chronic impact of recurrent illness. Although our primary analyses rigorously adjusted for numerous confounding variables, the possibility of residual confounding—particularly in differential expression analyses—should be considered.

In conclusion, our study provides a comprehensive transcriptomic analysis of a critical cortical (sACC) and subcortical (amygdala) brain region involved in emotion processing and MDD pathophysiology. We present consistent evidence for synaptic dysregulation across independent methods of analysis and highlight a genetically mediated dysregulation of synaptic and vesicular genes in excitatory cells that warrants further mechanistic inquiries and may have potential as future therapeutic targets. As emerging single-cell and long-read RNA sequencing technologies become feasible, future research can further clarify the specificity of the cellular and isoform-based mechanisms highlighted by this study.

## Methods

### Sample collection

Samples presented in this study were provided to the Lieber Institute for Brain Development Human Brain Repository from the State of Maryland and the Kalamazoo County, Michigan Medical Examiners’ Offices. Additional control subjects were also procured through the Gift of Life Michigan organ donation program. All the brain donations were performed under the auspices of the Western Institutional Review Board (WIRB), now known as WIRB-Copernicus Group IRB (study number 1126332). Informed consent was obtained from the next of kin, who also provided diagnostic information through a structured interview within 1 week of donation. Clinical and demographic data (shown in Supplementary Dataset 2) were obtained from all available medical and psychiatric records, and a comprehensive psychiatric summary was made for each case, summarizing all the available data^79^. Two board-certified psychiatrists independently review each case, reaching a consensus on DSM-IV/V Axis I lifetime diagnoses. Controls underwent an identical evaluation and psychiatric review, including a detailed next of kin interview screening for psychiatric symptoms, substance abuse, neurological and medical disorders. Both cases and controls underwent the same postmortem toxicology, neuropathology, and medication analyses.

The hemispheres from each postmortem brain were separated after detaching the brainstem, sliced into 1.5 cm coronal sections, flash-frozen, and stored at −80 °C. We dissected the cortical gray matter from the sACC from the medial aspect of the forebrain, ventral to the corpus callosum, and dorsal to the orbital frontal cortex using a handheld dental drill. Its boundaries are the interhemispheric fissure medially and the corona radiata/centrum semiovale laterally. The amygdala was dissected from the medial temporal lobe just anterior to the hippocampus, ventral to the anterior commissure and claustrum, and medial to the entorhinal cortex in order to include all subnuclei. Dissections were performed without knowledge of each brain’s case/control status, ensuring unbiased downstream analyses.

Sample selection for the current study included only subjects of European American ancestry. Across all samples, the mean RIN was 7.1 (±0.8 S.D.), the mean PMI was 29.2 h (±11.4 S.D.), and the mean age at death was 47.7 years (±16.4 S.D.). There were differences between cases and controls in terms of RIN (mean values 6.9 vs. 7.4), age at death (mean ages 44.4 vs 51.4), and sex (19% vs 33% females), but not in PMI. We therefore included RIN, age, and sex as covariates in our downstream gene expression analyses. Cases were selected based on a DSM-IV diagnosis of MDD with recurrent episodes. Consistent with the severity of the MDD phenotype, most cases had a lifetime history of suicidal ideation (78.8%) and past suicide attempts (55.4%). Approximately a third of the sample (*N* = 69, or 30%) died by suicide.

### RNA sequencing and analyses

RNA was extracted from dissected tissues, and sequencing libraries were prepared using the Illumina TruSeq Stranded Total RNA Ribo-Zero Gold protocol to deplete cytoplasmic and mitochondrial ribosomal RNA. Individual libraries were attached to multiplexed paired-end adapters, and six samples were sequenced per lane of an Illumina HiSeq 3000. Quality control (QC) was initially performed with FastQC (www.bioinformatics.babraham.ac.uk/projects/fastqc), followed by alignment the GENCODE release 25 (GRCh38.p7) human reference genome using the splice-aware aligner, HISAT2 version 2.0.4^80^. Counts were summarized at the level of the gene and exon using featureCounts v1.5.0-p3^81^, and quantification of junctions and transcripts was performed using regtools v. 0.1.0^82^ and Salmon v0.7.2^26^, respectively. External RNA Controls Consortium transcripts were included in each sequencing run and quantified with Kallisto version 0.43.0^83^.

### Quality control measures

After performing the preprocessing pipeline described above, we applied additional QC steps, leading to the exclusion of 41 samples for the reasons described below. First, we performed genotype-based principal component analyses and dropped 12 samples from six individuals of non-European American ancestry. Second, we excluded eight samples based on poor alignment metrics (overall mapping rate < 0.5, gene assignment rate < 0.3, and number of reads < 10 million) as shown in Supplementary Fig. 4. Third, we called genotypes using BCFtools and compared 740 common variants from the RNA sequencing results with our microarray-based genotypes to identify potential sample swaps. This led to the identification of 43 switched samples whose correct identities could be resolved and 10 samples that remained ambiguous and were dropped. Finally, we performed a further test for sample switching by identifying the 100 most differentially expressed genes between the sACC versus the Amygdala, resulting in the dropping of 11 outlier samples with gene expression signatures inconsistent with their labeled brain region (Supplementary Fig. 4). Following these QC steps, we explored and quantified the effect of potential confounders on gene expression by using the package variancePartition^84^ (Supplementary Fig. 6). Overall, and as shown in Supplementary Figs. 4 and 5, we found no to very small effects based on sequencing plates, ancestry-based principal components, or clinical comorbidity.

We subsequently normalized expression levels to reads per kilobase of feature per million mapped reads (RPKM) for genes and exons, reads per 10 million mapped reads (RP10M) for junctions, and transcripts per million (TPM) for transcripts. Low frequency features were removed using the expression cutoff() function from the Jaffelab R package using the following mean expression thresholds: 0.21 RPKM for genes, 0.24 RPKM for exons, 0.28 RP10M for junctions, and 0.30 TPM for transcripts.

### Quality surrogate variable analysis (qSVA)

We used quality surrogate variables (qSVs) to account for potentially latent RNA quality^85^ that may affect cases and controls differentially. As described in ref. ^51^, we performed tissue degradation experiments in tissue dissected from sACC and amygdala from five control donors from both sexes (two males and three females). Each dissection created four tissue aliquots that were left at room temperature for 0, 15, 30, and 60 min. RNA was extracted, sequenced, and processed as above, resulting in 20 RNA-seq samples in each brain region (five donors and four degradation time points). As expected, RINs dropped from a mean of 7.74 to 6.06 in the amygdala and from 8.36 to 7.3 in the sACC across this tissue degradation experiment. We subsequently combined all 40 RNA-seq samples to identify genomic regions associated with degradation across these two brain regions to calculate qSVs in our larger target dataset, allowing for a direct comparison of differentially expressed genes across the two brain regions. We fit a linear model to each expression region as a function of degradation time, adjusting for brain region and donor as fixed effects, and extracted the top 1000 degradation-susceptible regions. These regions were extracted from our larger case-control dataset for surrogate variable analyses^86^ that yielded 22 principal components, or qSVs, that were used as covariates in all downstream case-control analyses.

### SNP genotyping

Genotyping comprised several iterations of the Illumina Omni arrays (ranging from 1 million to 5 million assays) that were combined, lifted to GRCh38 Genome Reference Consortium Human Build 38 (hg38) coordinates, and cleaned using standard QC measures. Using Plink1.9^87^ we initially excluded variants with a minor allele frequency (MAF) less than 5%, a missingness rate of 5% or more, and a Hardy-Weinberg equilibrium *p* value < 1 × 10^−6^. We subsequently performed imputation using the TOPMED hg38 reference panel^88^, followed by a second round of similar QC measures, yielding a final filtered file consisting of 8,527,701 common variants.

### Cell-type deconvolution

To characterize the effect of cell-type composition, we utilized single-cell data from the Brain Initiative Cell Census Network (BICCN)^89^ to perform statistical deconvolution of our bulk RNA-seq dataset. We downloaded single-nucleus RNA data (https://cellxgene.cziscience.com/collections/) from the anterior cingulate cortex and the amygdala (central nuclear group) and utilized the program Hybrid-Scale Proportions Estimation (hspe, a recent extension of dtangle)^18^ to estimate cellular proportions in our bulk RNA-seq dataset. In a recent benchmark of several deconvolution algorithms^76^, we found the deconvolution algorithms in hspe and Bisque^90^ to be the most accurate among the commonly available deconvolution methods. In the current analyses, we used hspe rather than Bisque because of the acknowledged limitation of Bisque when snRNA donor sample sizes are small^90^. We used the cell clusters provided by BICNN, excluding rare clusters with  fewer than 100 cells sequenced. Cell-type proportion estimates (Supplementary Dataset 3) were used to compare case-control results and were considered to be significant following correction for multiple testing by brain region (shown in Supplementary Fig. 3).

### Differential gene and transcript expression analyses

We first normalized raw counts at the level of the gene to reads per kb of feature per million mapped reads (RPKM). Differential expression was subsequently tested using the limma package with voom transformation^91^ controlling for the following potential confounders:

$$Y=	 {{{\rm{Diagnosis}}}}+{{{\rm{Age}}}}+{{{\rm{Sex}}}}+{{{\rm{RIN}}}}+{{{\rm{mitochondrialRate}}}}+{{{\rm{rRNA}}}}\; {{{\rm{rate}}}} \\ 	+{{{\rm{total}}}}\; {{{\rm{Assigned}}}}\; {{{\rm{Gene}}}}+{{{\rm{abs}}}}\left({{{\rm{ERCCsumLogErr}}}}\right),\,+{{{{\rm{snpPCs}}}}}_{1{to}5} \\ 	+{{{\rm{quality}}}}\; {{{\rm{SurrogateVariables}}}}{\left({{{\rm{qSVAs}}}}\right)}_{1{to}22}$$

We accounted for multiple testing by controlling the false discovery rate (FDR) via the Benjamini–Hochberg algorithm and used FDR < 5% to declare findings genome-wide significant.

In transcript-based analyses, we used TPM (transcripts per million) output from Salmon v0.7.2 directly in Limma’s linear mixed model. We used the same covariates as in the gene-based analyses and similarly controlled for multiple testing using an FDR < 5% threshold.

### Differential splicing analyses

We utilized LeafCutter^28^ to quantify splicing patterns at the level of the gene. Beginning with junction counts produced by regtools (see above), we used LeafCutter to generate intron cluster counts requiring 50 split reads to support each cluster (*m* = 50) and allowing introns of up to 500 kb (*l* = 500,000). Combining the sACC and amygdala samples, LeafCutter generated 41,823 clusters with a total of 152,037 unique introns. Case-control analyses (differential intron excision analysis) were performed by brain region, applying a Dirichlet-multinomial generalized linear model using the same covariates from our expression-based differential expression analyses, followed by FDR correction for multiple testing. Differentially spliced intron associations with an FDR *p* value < 0.05 were subsequently filtered using a ΔPSI ≥ 1% threshold.

### Gene and synaptic ontology enrichment

We grouped genes or features significantly associated with MDD at the FDR < 5% level and used clusterProfiler^3^ to identify GO genesets (inclusive of the biological processes [“BP”], cellular component [“CC”], and molecular function [“MF”] sub-ontologies) that were significantly enriched (FDR < 5%). As background genes, we utilized all genes included in our differential expression analysis with an annotated Entrez ID (*n* = 23,468). To reduce redundancy across genesets and improve interpretability, we utilized the “simplify” function in clusterProfiler with a similarity cutoff value of 0.5.

For a more detailed evaluation of synaptic enrichment, we utilized SynGo version 1.2 (https://www.syngoportal.org/)^57^ to perform enrichment analyses. We combined all associated genes in our differential expression analyses at the level of the gene, transcript, and splice cluster for each brain region and used the combined background genes from these three features as our testing background. For QTL analyses, we similarly tested a combined set of all genes per region with significant analyses in our gene (TWAS, SMR, coloc), splice (SMR, coloc), and transcript (SMR, coloc) integrative analyses (described below). As background for these genesets (one per brain region), we utilized the same background list from the combined (gene, transcript, and LeafCutter) differential expression analyses.

### Weighted gene co-expression network analysis (WGCNA)

Using the gene-level count data from both cases and controls in both brain regions we analyzed them independently to identify region-specific modules of co-expressed genes. We initially residualized our normalized gene counts from the covariates used in our case-control analyses (see above) and ran WGCNA^92^ with the following parameters: networkType = “signed,” minModuleSize = 50, corType = “bicor,” mergeCutHeight = 0.2, deepSplit = 4, maxBlockSize = 30,000.

We tested whether each module was associated with case-control status by extracting the first expression-based principal component from each module (“module eigengene”) and using FDR correction to account for multiple testing. Each module was subsequently characterized for GO enrichment terms using clusterProfiler. Using a Fisher exact test, we tested each module for enrichment without differentially expressed genes and with the 411 genes found to be genome-wide significant in the Als et al. MAGMA gene-based analyses (Als et al.^4^). To compare the pattern of GWAS-associated gene-based enrichments across associated phenotypes, we also used the gene-based (MAGMA) results from GWAS meta-analyses of PTSD^93^, alcohol use disorder^94^, and bipolar disorder^95^.

We also tested for cell-type enrichments of implicated modules with cell-type-specific genes for each reported cell type (astrocytes, endothelial cells, excitatory neurons, inhibitory neurons, microglia, oligodendrocytes, oligodendrocyte progenitor cells, T-cells) defined based on the Tran et al. single-nucleus RNA-seq data^96^. Association testing was corrected using FDR < 5% within each type of enrichment analysis.

### Expression, splicing, and transcript-based quantitative trait loci (QTL) analyses

Using tensorQTL^52^, we modeled the gene, transcript, and normalized intron excision counts (“splicing QTLs”) as a function of SNP genotypes using linear regression to identify candidate expression quantitative trait loci (eQTLs) in each brain region. All models controlled for diagnosis, sex, the top principal components of the expression data at each feature level to adjust for potential technical artifacts, and five SNP-based principal components. QTL analyses were performed using a ±500 kb window around markers with an MAF > 5%. QTLs analysis was performed genome-wide, followed by FDR correction within each of the transcription features (gene, transcript, and local splicing).

### Integrative locus-based analyses

Co-localization of the MDD genome-wide significant loci^4^ was performed using COLOC^8^. For each lead variant in significant loci, we initially converted the base pair coordinates to hg38 using the UCSC liftOver tool, extracted all overlapping variant-feature associations from the eQTL results that overlapped with the imputed MDD GWAS association results^7^. We subsequently extracted region-specific eGenes within each locus defined as ±500 Kb around each of the  243 GWAS-significant lead variants. We subsequently tested these region-specific eGenes using COLOC, focusing on the posterior probability testing the hypothesis of co-localization (PP.H4) between the MDD GWAS signal and the region-specific eQTL association. As a complement to these analyses, we also performed SMR^46^ of the same genome-wide loci. We used a Bonferroni correction for the number of genes tested in the SMR analyses and prioritized those with an SMR HEIDI (Heterogeneity in Dependent Instruments) *P* value ≥ 0.05.

### Transcriptome-wide Association Study (TWAS)

We used the TWAS approach by Gusev et al.^44^ to compute feature weights at the gene level separately in the sACC and amygdala, and to integrate these with MDD GWAS statistics from Als et al.^4^. As recommended by the original developers of TWAS^3^, we used a 500 kb window for each gene, filtered for an expression-based  heritability *P* value < 0.01. We subsequently applied expression-based gene weights to the MDD GWAS-based summary statistics and calculated TWAS association statistics using the FUSION.assoc_test.R script. The TWAS association was Bonferroni corrected for the number of genes with significant transcript-based heritability in each brain region.

### Reporting summary

Further information on research design is available in the Nature Portfolio Reporting Summary linked to this article.

## Supplementary information

Supplementary Information

Supplementary Dataset 1

Supplementary Dataset 2

Supplementary Dataset 3

Supplementary Dataset 4

Supplementary Dataset 5

Supplementary Dataset 6

Supplementary Dataset 7

Supplementary Dataset 8

Supplementary Dataset 9

Supplementary Dataset 10

Supplementary Dataset 11

Reporting Summary

Transparent Peer Review file

## Data Availability

Whole-genome (microarray) based genotypes and RNA sequencing data are available in the Synapse data platform under accession code 22276064. Genome-wide expression, splice, and transcript-based QTL analyses are available as a Zenodo dataset (https://zenodo.org/records/13906097).

## Code availability

Code for all analyses described here is available on GitHub:

https://github.com/LieberInstitute/goesHyde_mdd_rnaseq (10.5281/zenodo.15013040).
