# Supplementary material for: Large-scale transcriptomic analyses of major depressive disorder reveal convergent dysregulation of synaptic pathways in excitatory neurons
Source: Nat Commun. 2025 Apr 28;16:3981. doi: 10.1038/s41467-025-59115-4 (PMC12037741; doi:10.1038/s41467-025-59115-4)
Supplement: Supplementary file 1 — Supplementary Information [file 41467_2025_59115_MOESM1_ESM.pdf]

Supplementary Table 1: Summary of RNA sequencing Studies in Major Depressive Disorder

Study	*Pubmed ID*	*Year*	*Tissue*	*Sample*	*Assay/ Analysis*	*N per Group (MDD / Controls)*	
Kohen et al.	24594777	2014	Dentate Gyrus	Stanley Brain Collection / UW Neuropathology Brain bank	RNAseq /(ABI SOLiD 4) Multiple DGE analyses	17 / 29	
Kim et al.	26077692	2016	Hippocampus	Stanley Brain Collection	RNAseq	15 / 15	MDD vs Control 3 genes FDR < 5%
Darby et al.	27622934	2016	OFC	Stanley Brain Collection	RNAseq	15 / 15	MDD vs Control 16 genes FDR < 5%
Labonte et al.	28825715	2017	DLPFC, NAc, OFC, anterior insula, ACC, ventral subiculum	Douglas Mental Health Institute / UT Southwestern brain	RNAseq (sex-stratified) DGE/limma	26 / 22	Sex-stratified DGE (nominal P > 0.05)
Pantazatos et al.	27528462	2017	DLPFC	New York State Psychiatric Institute	RNAseq DGE/DESeq2	30 / 29	MDD vs Control 4 genes FDR < 5%
Ramaker et al.	28754123	2017	DLPFC, ACC, NAc	University of California Irvine / Pritzker	RNAseq	24 / 24	MDD vs Control No gene FDR < 5%
Mahajan et al.	29175309	2018	Dentate Gyrus	Cuyahoga County Medical Examiner's Office	RNAseq DESeq2	23 / 23	MDD vs Control 30 genes FDR < 5%
Girgenti et al.	33349712	2021	DLPFC, OFC, dACC, sACC	National Center for PTSD Brain Bank / University Pittsburg	RNAseq DGE/DESeq2	45 / 46	sACC MDD vs Con (4065 genes FDR < 5%)
Akula et al.	33349712	2021	sACC	NIMH Human Brain Bank	RNAseq DGE/DESeq2	51 / 55	MDD vs Control (7 genes FDR < 5%)
Shukla et al	34686766	2022	PFC	Allegheny County Medical Examiner's Office	RNAseq DGE/DESeq2	70 /20	MDD(all) vs Control No FDR < 5%
Mansouri et al	37884562	2023	DLPFC, NAc, OFC, anterior insula, ACC, ventral subiculum	Douglas Mental Health Institute / UT Southwestern brain	RNAseq (sex-stratified) DGE (limma) (inclusive of Labonte et al.)	42/47	Sex-stratified DGE (nominal P > 0.05)

Tissue abbreviations:

OFC: Orbitofrontal Cortex
DLPFC: Dorsolateral Prefrontal Cortex
NAc: Nucleus Accumbens
Anterior Insula: Anterior Insular Cortex (dorsal or subgenual)
ACC: Anterior Cingulate Cortex
Ventral Subiculum: Ventral Subicular Complex

Supplementary Figure 1: Distribution and Overlap of Differentially Expressed Genes in RNAseq studies of MDD

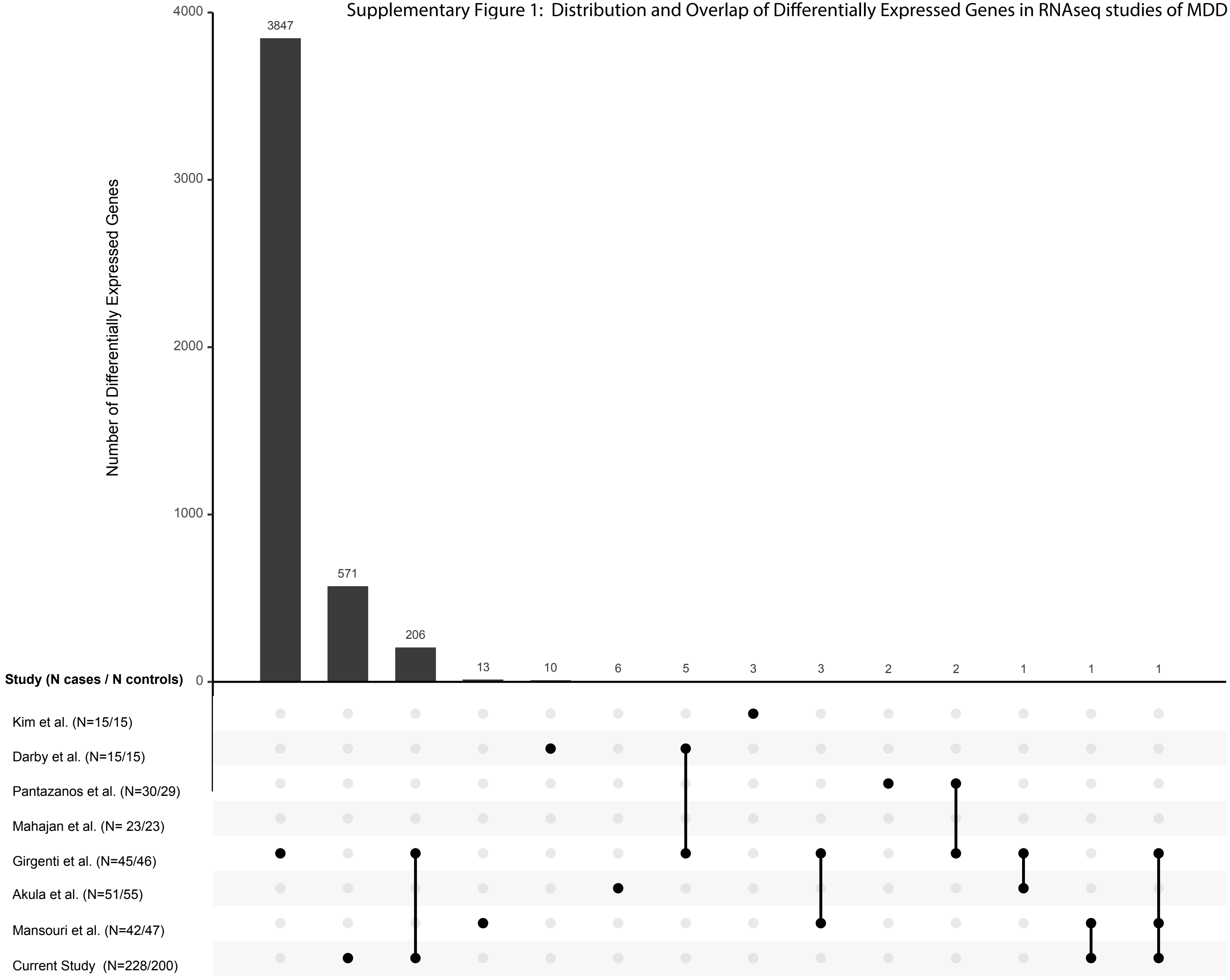

Distribution and Overlap of Differentially Expressed Genes in RNAseq studies of the MDD post-mortem brain

Supplementary Figure 2: Polygenic risk scores by case–control status (A) and stratified by sex (B).

A

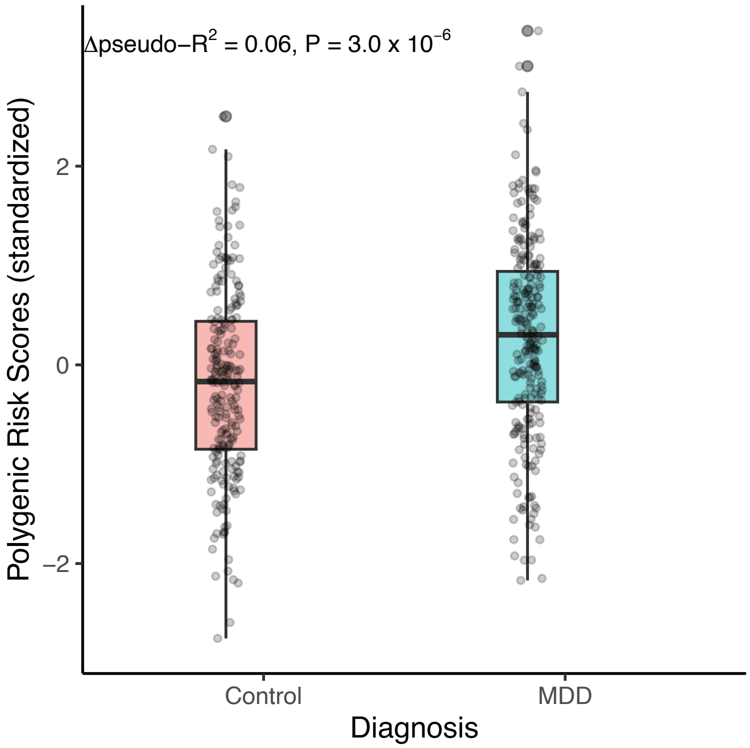

B

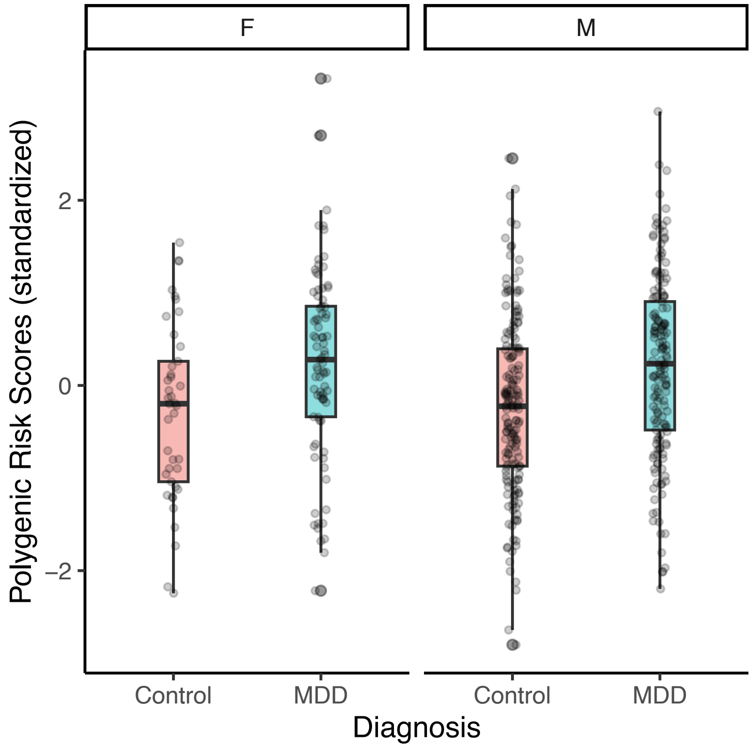

Polygenic risk scores by case–control status (A) and stratified by sex (B).
The top figure additionally shows the proportion of variance explained by case control status (ΔR2) and the P-value of the case-control regression coefficient.

Supplementary Figure 3: Cellular Deconvolution Proportions (significant differences highlighted)

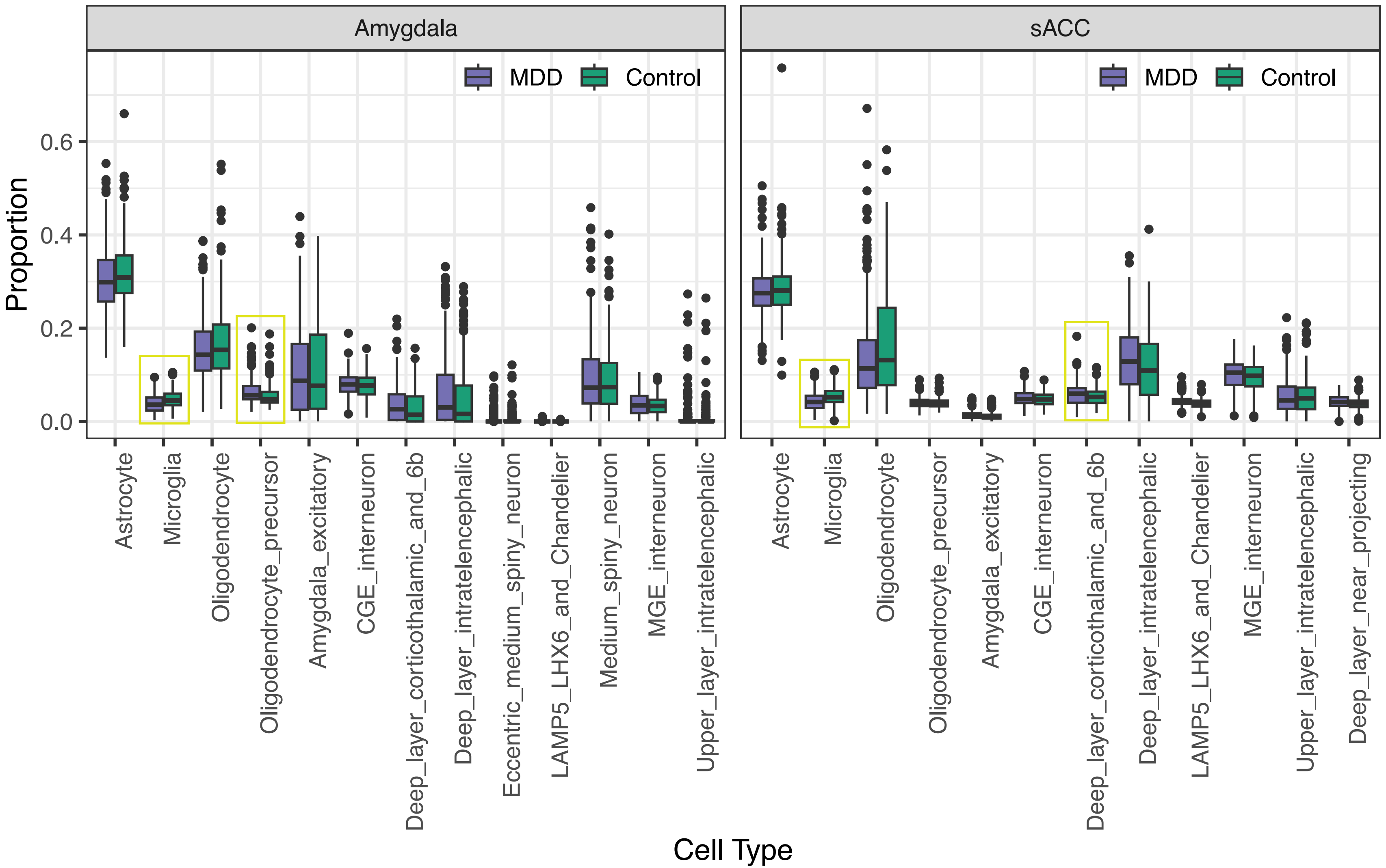

Cellular Deconvolution Proportions on the BRAIN Initiative - Cell Census Network (BICCN) classification using the hspe (hybrid-scale proportions estimation) package. Proportions are plotted by brain region and compared between MDD cases and controls. Proportions with significant case–control differences are highlighted in yellow.

Supplementary Figure 4: Quality Control Metrics

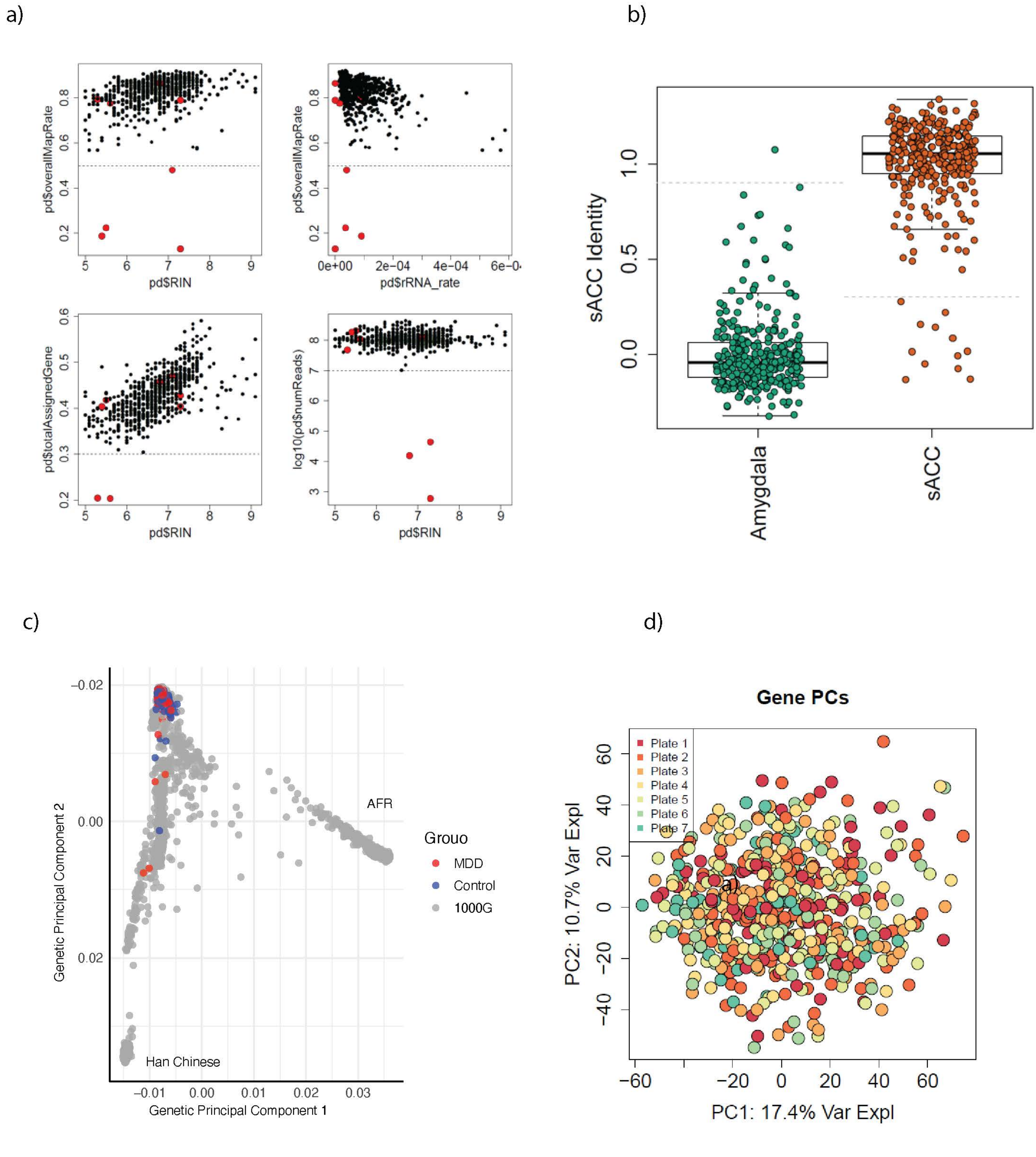

Panel A: Distribution of samples across key QC metrics from Supplementary Dataset 2 (RIN, overall mapping rate, percentage of ribosomal RNA, proportion of reads mapping to genic regions, and total number of reads). Dotted lines indicate boundary thresholds for outlier exclusion.
Panel B: Distribution of samples showing region-specific identity, determined by region-specific genes. Dotted lines indicate samples excluded during quality control.
Panel C: Gene-based principal component analysis, anchored by samples from the 1000 Genomes Project.
Panel D: Distribution of RNA-seq–based expression principal components, showing no evidence of batch effects by sequencing plates.

Supplementary Figure 5: Distribution of Post-Mortem Interval by case-control Status

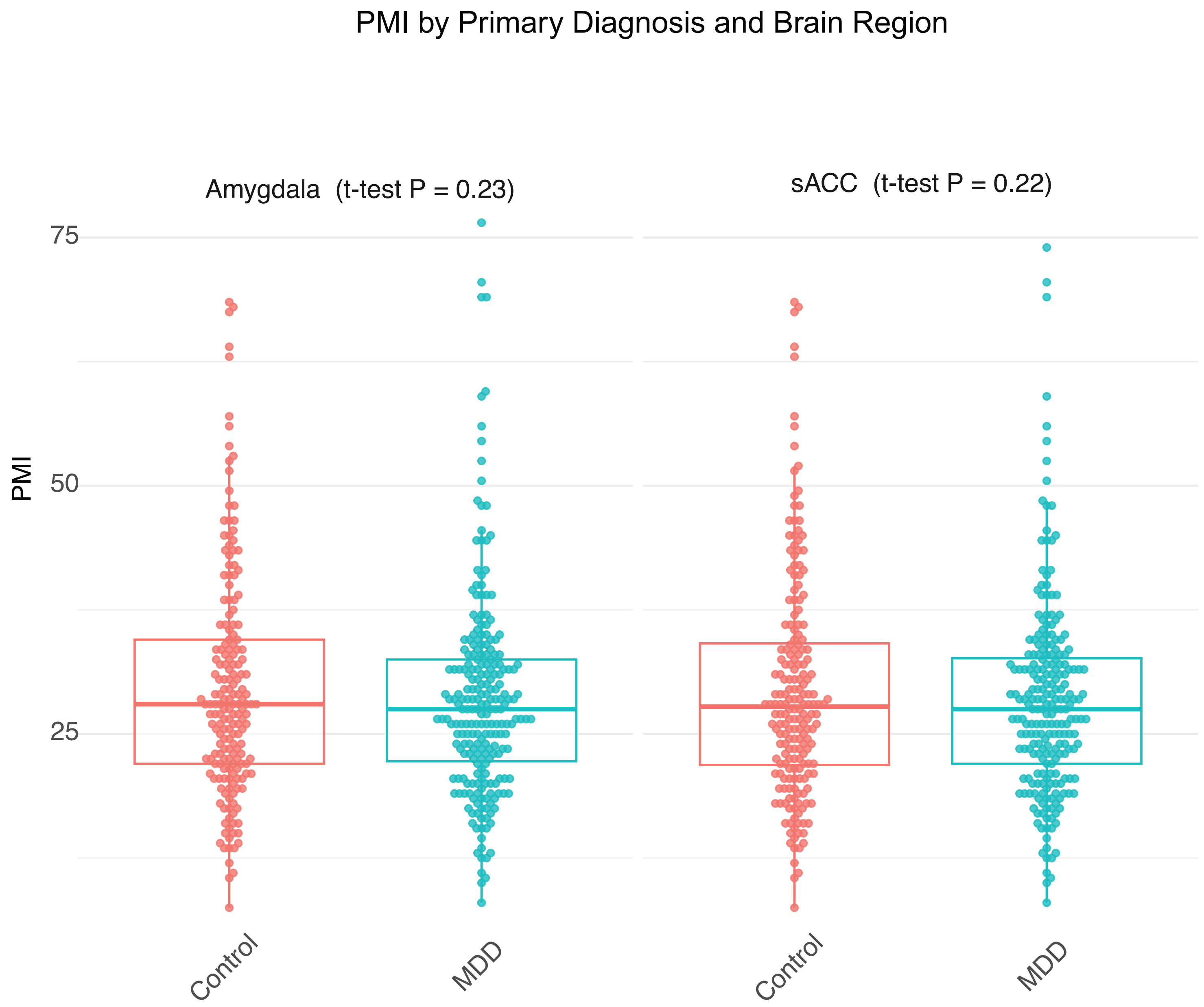

Distribution of post-mortem interval (PMI) by case–control status in the amygdala and sACC. Comparisons with t-test shows no significant difference in PMI between cases and controls.

Supplementary Figure 6: Variance Partitioning Plot of Gene Expression Counts

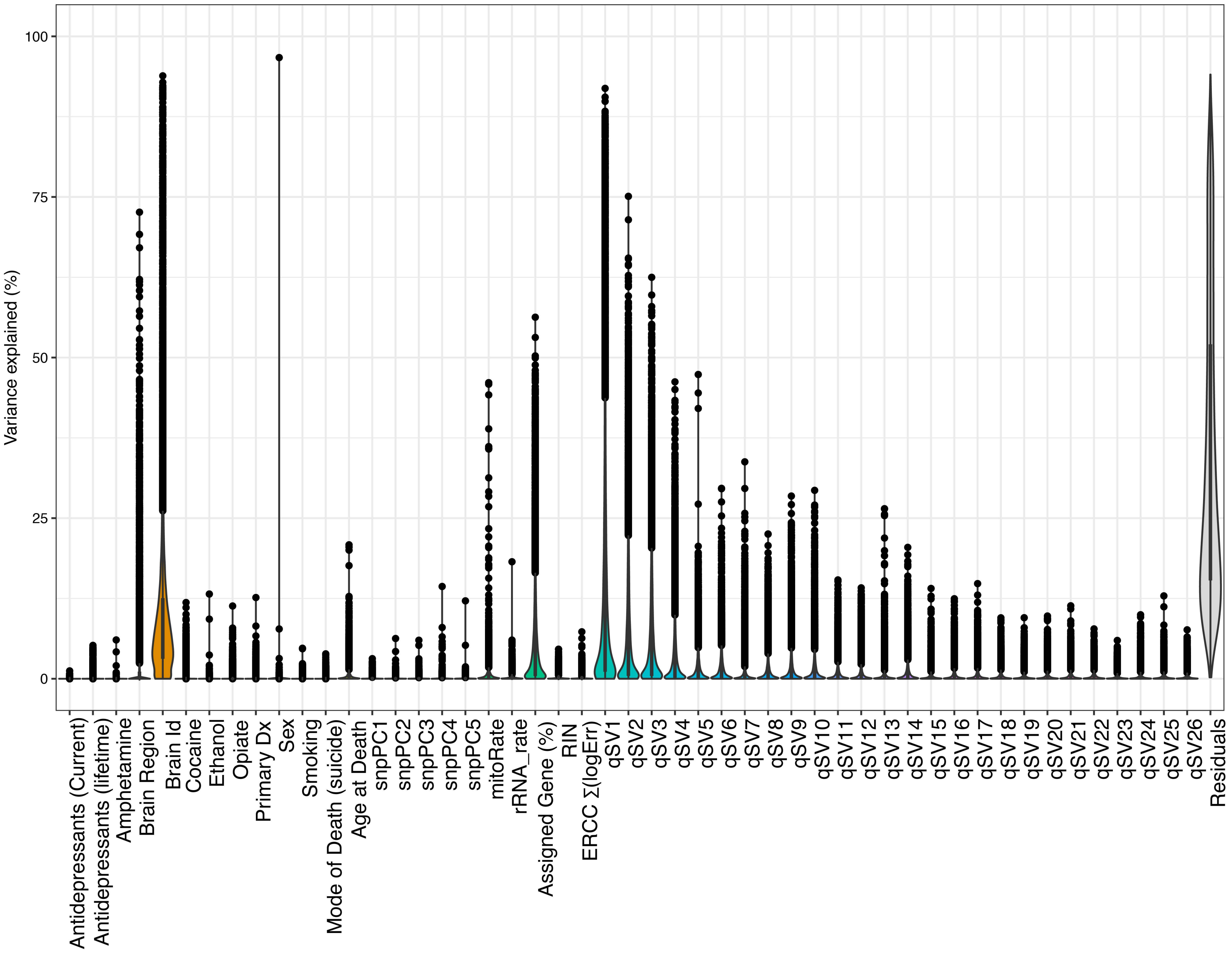

Variance partitioning of gene expression by technical and clinical covariates using the variancePartition R package

Supplementary Figure 7a: Correlation Matrix of Covariates used in the sACC

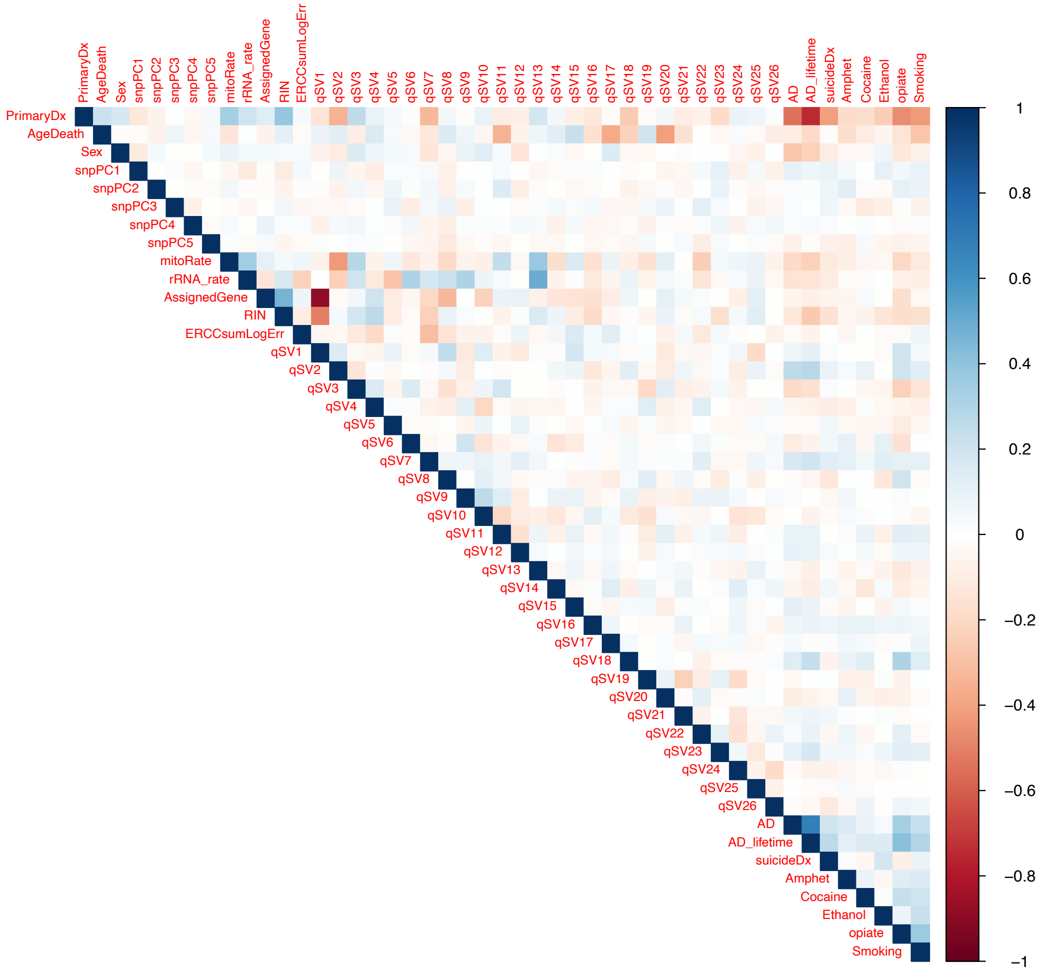

Supplementary Figure 7b: Correlation Matrix of Covariates used in the Amygdala

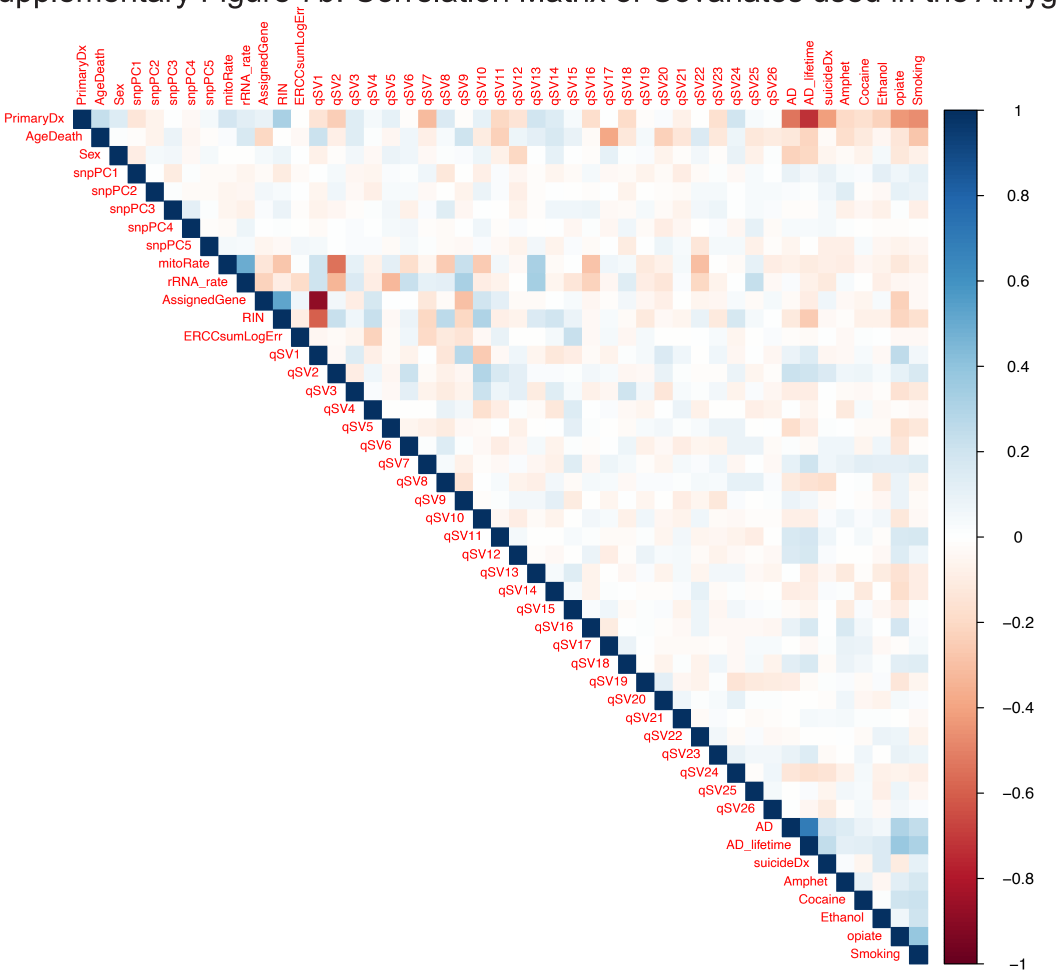

Correlation matrix of technical, clinical and genetic covariates included in the analyses of the (a) sACC and (b) amygdala

Supplementary Figure 8a:

Comparison of Differential Gene Expression $\log_2$(Fold Change) in the sACC versus the amygdala

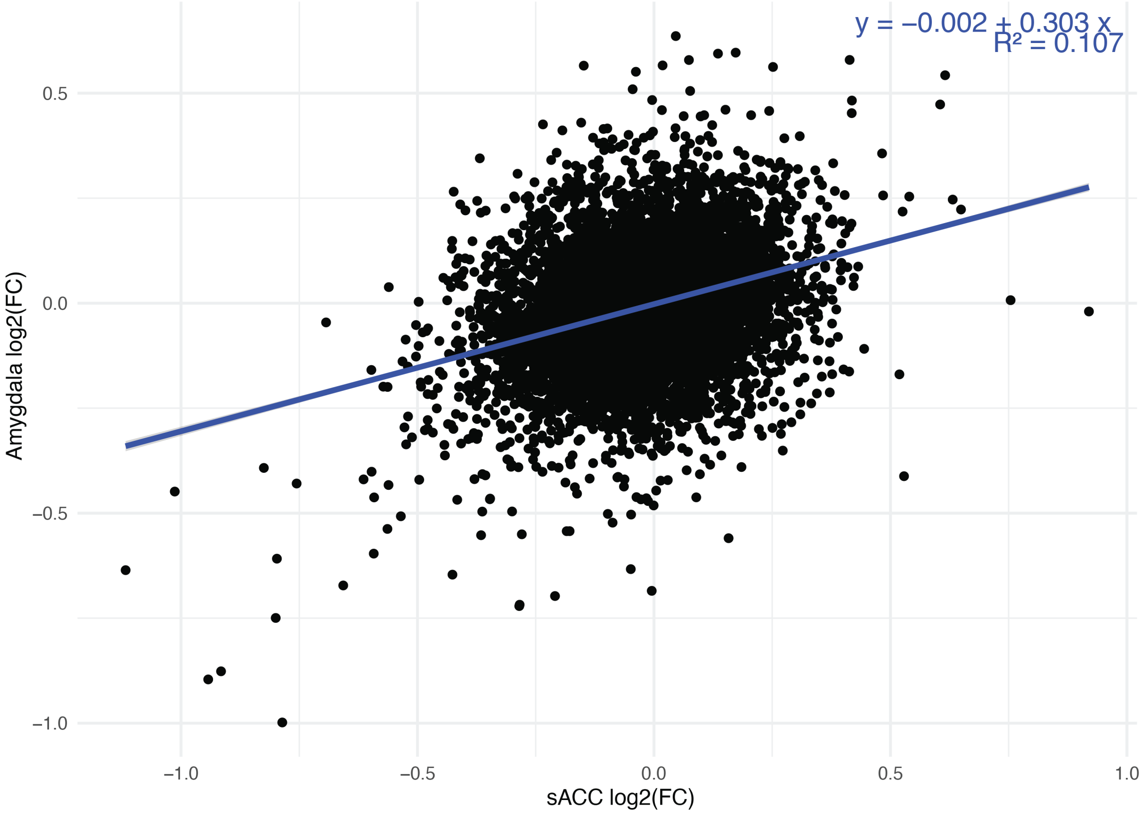

Supplementary Figure 8b:

Comparison of Differential Transcript Expression $\log_2$(Fold Change) in the sACC versus the amygdala

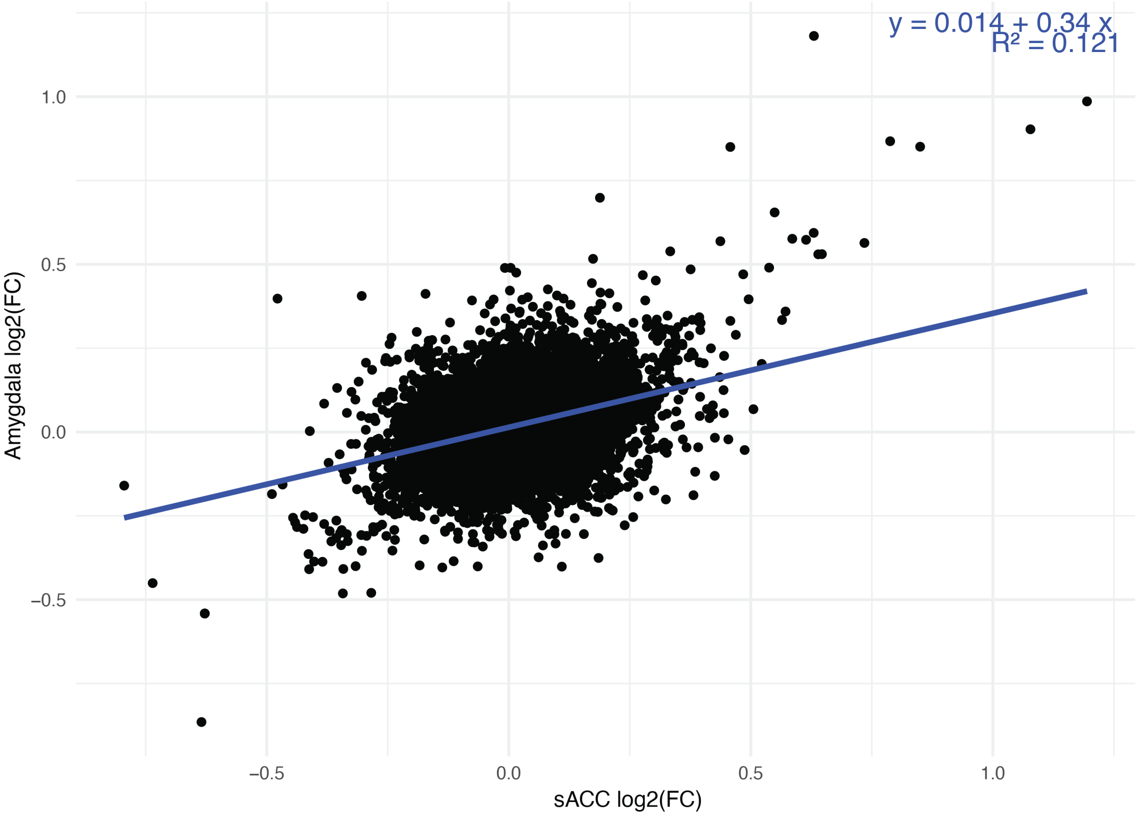

Equations represents parameters estimated by the linear regression model (β coefficients and intercept), along with the coefficient of determination (R^2) indicating model fit.

Supplementary Figure 9: Distribution Quantitative Trait Loci relative to the Transcription Start Site

Gene QTL

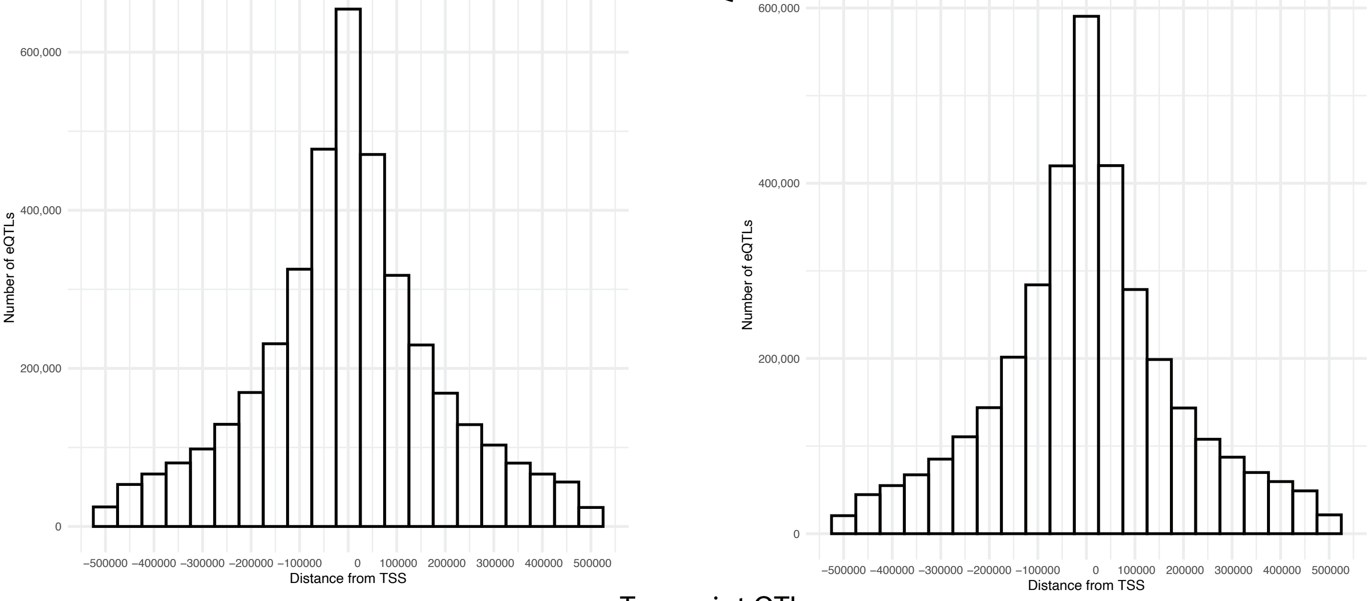

Transcript QTL

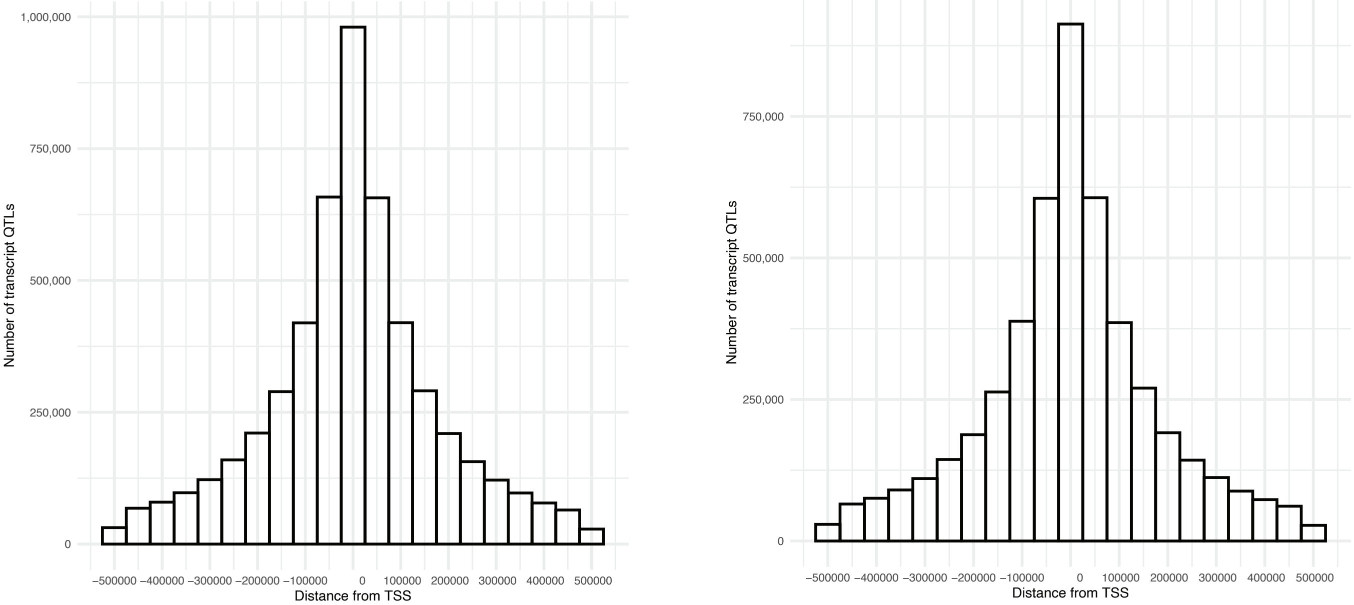

Splice QTL

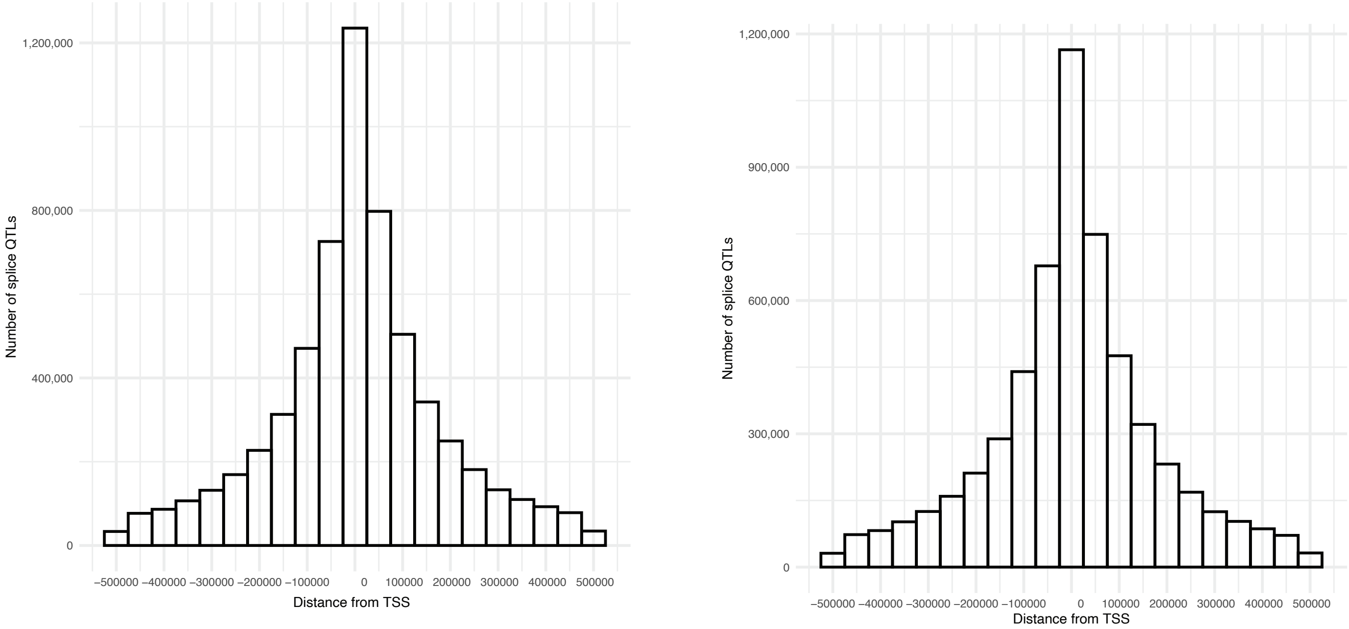

Distribution of quantitative trait loci (QTLs) relative to the transcription start site for gene-, transcript-, and splice-level QTLs in the sACC (left) and amygdala (right)

Supplementary Figure 10a: Overlap of TWAS/QTL associated transcriptional features in the sACC

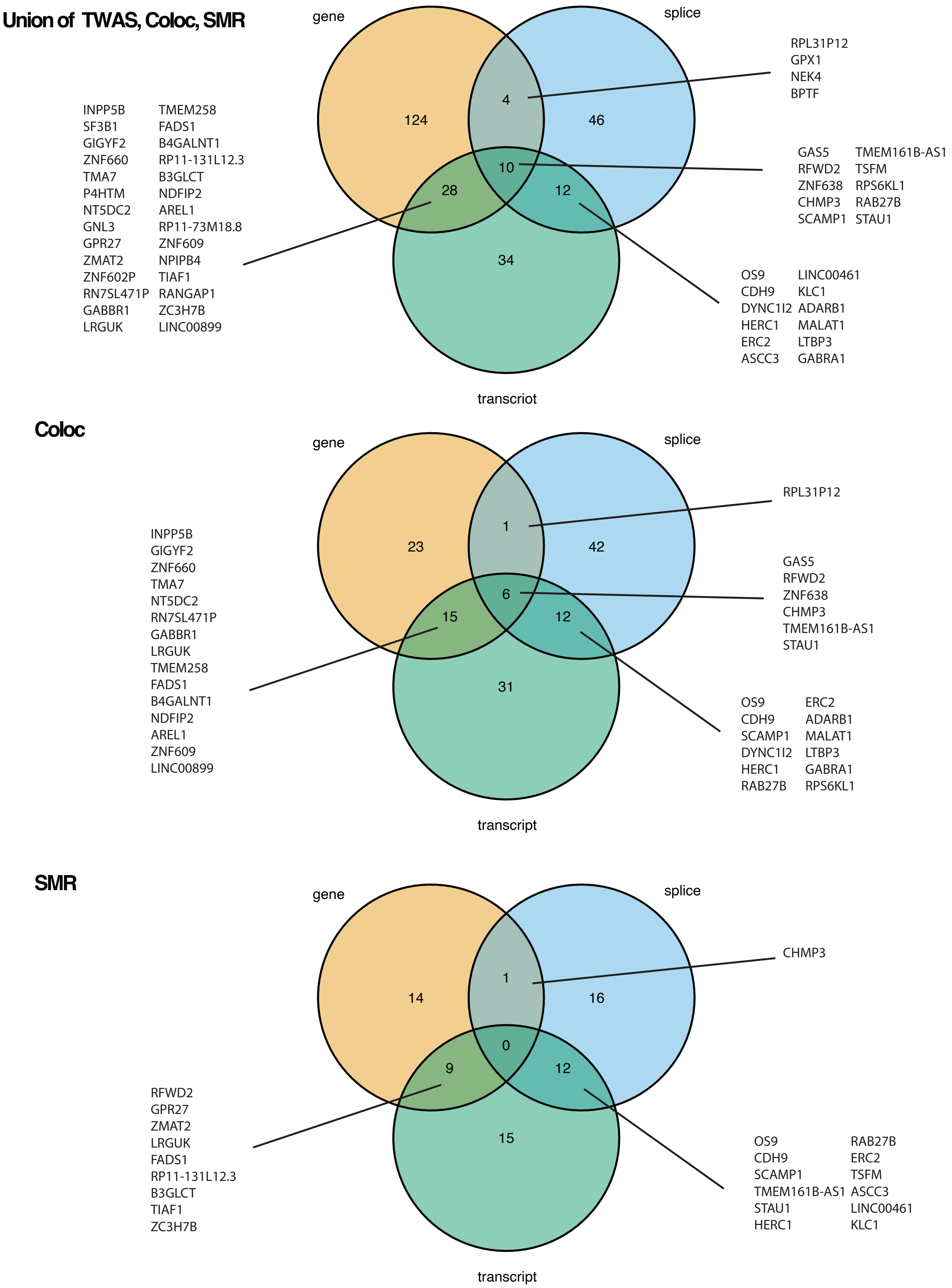

The top subfigure shows the overlap of genes, splicing clusters, and transcripts identified as significant in TWAS, coloc, or summary-based Mendelian Randomization (SMR) analyses. The middle subfigure highlights significant genes, splicing clusters, and transcripts in coloc analyses (PP.H4 > 0.8), while the bottom subfigure displays the overlap for SMR analyses (marker–feature associations with a Bonferroni-corrected p-value < 0.05).

Supplementary Figure 10b: Overlap of TWAS/QTL associated transcriptional features in the amygdala

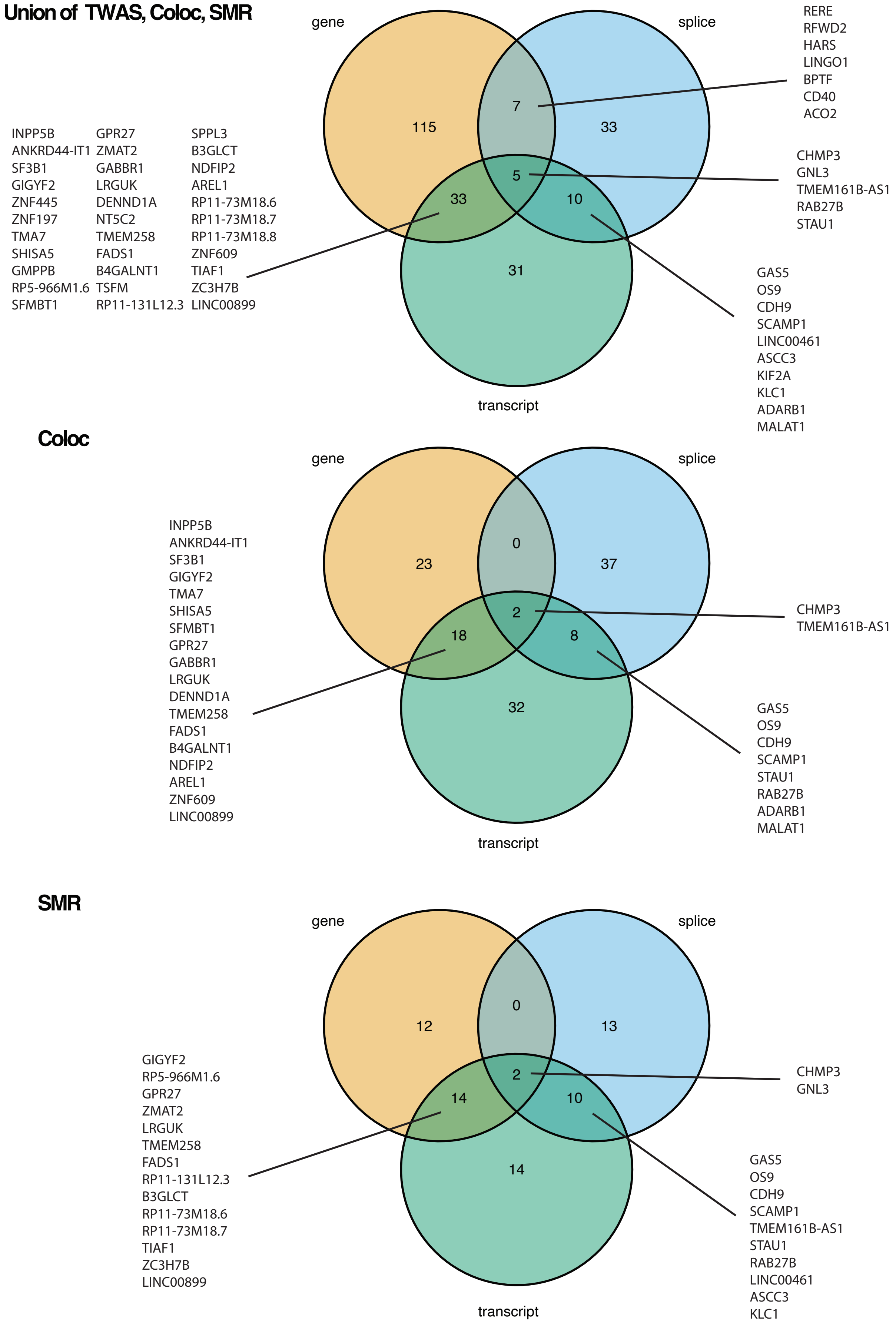

The top subfigure shows the overlap of genes, splicing clusters, and transcripts identified as significant in TWAS, coloc, or summary-based Mendelian Randomization (SMR) analyses. The middle subfigure highlights significant genes, splicing clusters, and transcripts in coloc analyses (PP.H4 > 0.8), while the bottom subfigure displays the overlap for SMR analyses (marker–feature associations with a Bonferroni-corrected p-value < 0.05).
