# Supplementary material for: Large-scale transcriptomic analyses of major depressive disorder reveal convergent dysregulation of synaptic pathways in excitatory neurons
Source: Nat Commun. 2025 Apr 28;16:3981. doi: 10.1038/s41467-025-59115-4 (PMC12037741; doi:10.1038/s41467-025-59115-4)
Supplement: Supplementary file 13 — Reporting Summary [file 41467_2025_59115_MOESM13_ESM.pdf]

nature portfolio

Corresponding author(s): Fernando S. Goes and Thomas Hyde

Last updated by author(s): Feb 12, 2024

Reporting Summary

Nature Portfolio wishes to improve the reproducibility of the work that we publish. This form provides structure for consistency and transparency in reporting. For further information on Nature Portfolio policies, see our Editorial Policies and the Editorial Policy Checklist.

Statistics

For all statistical analyses, confirm that the following items are present in the figure legend, table legend, main text, or Methods section.

n/a	Confirmed	
☐	☒	The exact sample size (*n*) for each experimental group/condition, given as a discrete number and unit of measurement
☐	☒	A statement on whether measurements were taken from distinct samples or whether the same sample was measured repeatedly
☐	☒	The statistical test(s) used AND whether they are one- or two-sided<br>*Only common tests should be described solely by name; describe more complex techniques in the Methods section.*
☐	☒	A description of all covariates tested
☐	☒	A description of any assumptions or corrections, such as tests of normality and adjustment for multiple comparisons
☐	☒	A full description of the statistical parameters including central tendency (e.g. means) or other basic estimates (e.g. regression coefficient) AND variation (e.g. standard deviation) or associated estimates of uncertainty (e.g. confidence intervals)
☐	☒	For null hypothesis testing, the test statistic (e.g. *F*, *t*, *r*) with confidence intervals, effect sizes, degrees of freedom and *P* value noted<br>*Give P values as exact values whenever suitable.*
☐	☒	For Bayesian analysis, information on the choice of priors and Markov chain Monte Carlo settings
☐	☒	For hierarchical and complex designs, identification of the appropriate level for tests and full reporting of outcomes
☐	☒	Estimates of effect sizes (e.g. Cohen's *d*, Pearson's *r*), indicating how they were calculated

Our web collection on statistics for biologists contains articles on many of the points above.

Software and code

Policy information about availability of computer code

Data collection	No software was used in initial data collection
Data analysis	We have made the code used in our analyses available at: https://github.com/LieberInstitute/goesHyde_mdd_rnaseq

For manuscripts utilizing custom algorithms or software that are central to the research but not yet described in published literature, software must be made available to editors and reviewers. We strongly encourage code deposition in a community repository (e.g. GitHub). See the Nature Portfolio guidelines for submitting code & software for further information.

Data

Policy information about availability of data

All manuscripts must include a data availability statement. This statement should provide the following information, where applicable:

- Accession codes, unique identifiers, or web links for publicly available datasets
- A description of any restrictions on data availability
- For clinical datasets or third party data, please ensure that the statement adheres to our policy

Whole genome microarray based genotypes and RNA sequencing data is available in the synapse data platform under accession code 22276066
EQTL data is publically avaialable at : https://zenodo.org/records/13906097

Research involving human participants, their data, or biological material

Policy information about studies with human participants or human data. See also policy information about sex, gender (identity/presentation), and sexual orientation and race, ethnicity and racism.

Reporting on sex and gender	The sample included 615 samples from male individuals (72.7%) and 231 from female individuals.
Reporting on race, ethnicity, or other socially relevant groupings	To reduce the risk of residual population stratification, all samples were initially identified as White and of European-American descent by their next of kin
Population characteristics	Samples were exclusively from individuals of European American ancestry
Recruitment	Samples were collected from the Office of the Chief Medical Examiner for the State of Maryland or the Kalamazoo County Michigan Medical Examiners'Office.
Ethics oversight	The samples were collected with consent from next of kin. The collection of the samples from the various locations were approved under the State of Maryland Department of Health and Mental Hygiene Protocol 12-24, and under Western Institutional Review Board Protocol 20111080.

Note that full information on the approval of the study protocol must also be provided in the manuscript.

Field-specific reporting

Please select the one below that is the best fit for your research. If you are not sure, read the appropriate sections before making your selection.

☒ Life sciences ☐ Behavioural & social sciences ☐ Ecological, evolutionary & environmental sciences

For a reference copy of the document with all sections, see nature.com/documents/nr-reporting-summary-flat.pdf

Life sciences study design

All studies must disclose on these points even when the disclosure is negative.

Sample size	Sample sizes were selected to have 80% power to identify differentially expressed genes with a fold change of 1.09 and an eqtl slope beta effect of at least 0.13
Data exclusions	We excluded 8 samples based on poor read alignment metrics, 11 due to region discrepancy (consistent with a lab error), 10 due to ambiguous matching based on genotyping data and 12 samples that were of non-European ancestry on principal components.
Replication	We have compared our findings with other published findings, but were limited in a strict replication aim, since there no other comparable post-mortem studies in MDD.
Randomization	Samples were randomized to sequencing batches in order to minimize batch effects in our case-control analyses
Blinding	The investigators were blinded to case status during preparation of the tissue samples

Reporting for specific materials, systems and methods

We require information from authors about some types of materials, experimental systems and methods used in many studies. Here, indicate whether each material, system or method listed is relevant to your study. If you are not sure if a list item applies to your research, read the appropriate section before selecting a response.

Materials & experimental systems

n/a	Involved in the study	
☒	☐	Antibodies
☒	☐	Eukaryotic cell lines
☒	☐	Palaeontology and archaeology
☒	☐	Animals and other organisms
☒	☐	Clinical data
☒	☐	Dual use research of concern
☒	☐	Plants

Methods

n/a	Involved in the study	
☒	☐	ChIP-seq
☒	☐	Flow cytometry
☒	☐	MRI-based neuroimaging

Plants

Seed stocks

Report on the source of all seed stocks or other plant material used. If applicable, state the seed stock centre and catalogue number. If plant specimens were collected from the field, describe the collection location, date and sampling procedures.

Novel plant genotypes

Describe the methods by which all novel plant genotypes were produced. This includes those generated by transgenic approaches, gene editing, chemical/radiation-based mutagenesis and hybridization. For transgenic lines, describe the transformation method, the number of independent lines analyzed and the generation upon which experiments were performed. For gene-edited lines, describe the editor used, the endogenous sequence targeted for editing, the targeting guide RNA sequence (if applicable) and how the editor was applied.

Authentication

Describe any authentication procedures for each seed stock used or novel genotype generated. Describe any experiments used to assess the effect of a mutation and, where applicable, how potential secondary effects (e.g. second site T-DNA insertions, mosiacism, off-target gene editing) were examined.
